# Optimized continuous homecare provisioning through distributed data-driven semantic services and cross-organizational workflows

**DOI:** 10.1186/s13326-024-00303-4

**Published:** 2024-06-06

**Authors:** Mathias De Brouwer, Pieter Bonte, Dörthe Arndt, Miel Vander Sande, Anastasia Dimou, Ruben Verborgh, Filip De Turck, Femke Ongenae

**Affiliations:** 1 Department of Information Technology, IDLab – Ghent University – imec, 9052 Ghent, Belgium; 2 Stream Intelligence Lab, KU Leuven Kulak, Kortrijk, 8500 Belgium; 3 International Center for Computational Logic, Technische Universität Dresden, 01187 Dresden, Germany; 4 meemoo, Flemish Institute for Archives, 9000 Ghent, Belgium; 5 Department of Computer Science, KU Leuven, 2860 Sint-Katelijne-Waver, Belgium; 6 Department of Electronics and Information Systems, IDLab – Ghent University – imec, 9052 Ghent, Belgium

**Keywords:** Continuous homecare, Data-driven service, Distributed architecture, Cross-organizational workflows, Stream reasoning, Healthcare

## Abstract

**Background:**

In healthcare, an increasing collaboration can be noticed between different caregivers, especially considering the shift to homecare. To provide optimal patient care, efficient coordination of data and workflows between these different stakeholders is required. To achieve this, data should be exposed in a machine-interpretable, reusable manner. In addition, there is a need for smart, dynamic, personalized and performant services provided on top of this data. Flexible workflows should be defined that realize their desired functionality, adhere to use case specific quality constraints and improve coordination across stakeholders. User interfaces should allow configuring all of this in an easy, user-friendly way.

**Methods:**

A distributed, generic, cascading reasoning reference architecture can solve the presented challenges. It can be instantiated with existing tools built upon Semantic Web technologies that provide data-driven semantic services and constructing cross-organizational workflows. These tools include RMLStreamer to generate Linked Data, DIVIDE to adaptively manage contextually relevant local queries, Streaming MASSIF to deploy reusable services, AMADEUS to compose semantic workflows, and RMLEditor and Matey to configure rules to generate Linked Data.

**Results:**

A use case demonstrator is built on a scenario that focuses on personalized smart monitoring and cross-organizational treatment planning. The performance and usability of the demonstrator’s implementation is evaluated. The former shows that the monitoring pipeline efficiently processes a stream of 14 observations per second: RMLStreamer maps JSON observations to RDF in 13.5 ms, a C-SPARQL query to generate fever alarms is executed on a window of 5 s in 26.4 ms, and Streaming MASSIF generates a smart notification for fever alarms based on severity and urgency in 1539.5 ms. DIVIDE derives the C-SPARQL queries in 7249.5 ms, while AMADEUS constructs a colon cancer treatment plan and performs conflict detection with it in 190.8 ms and 1335.7 ms, respectively.

**Conclusions:**

Existing tools built upon Semantic Web technologies can be leveraged to optimize continuous care provisioning. The evaluation of the building blocks on a realistic homecare monitoring use case demonstrates their applicability, usability and good performance. Further extending the available user interfaces for some tools is required to increase their adoption.

**Supplementary Information:**

The online version contains supplementary material available at 10.1186/s13326-024-00303-4.

## Background

### Introduction

Due to increased digitization allowing more easily capturing relevant data, industries are faced with the challenge of processing an increase in complex and heterogeneous data in an automated, scalable, performant and cost-efficient manner. Increasing demand can also be noted for offering more personalized, context-aware and intelligent applications to end users [1, 2]. This translates into increased non-recurring engineering costs, a need to build custom interfaces, and a long time to market.

To tackle these issues, during the last years, companies increasingly adopt a Service Oriented Architecture (SOA) in which systems consist of services that each offer a self-contained unit of functionality [3]. By combining services into workflows, the required functionality can be offered in a structured way [4]. Existing workflow engines allow easily reusing services in different workflows, resulting in lower development and maintenance costs and a quicker time to market. Nevertheless, usually, custom APIs are built to expose each available data source, on which custom services are built that manually need to be configured into static workflows that fulfill a particular need. As a result, the intelligence of a platform is distributed over these different assets, making management an immense burden. Every time a new data source or service becomes available, manual configuration effort is required to integrate it and set up a cross-organizational workflow, leading to high costs, possible configuration errors, and custom APIs and services that cannot be reused. In addition, the custom services lack a personalized approach: available knowledge cannot be efficiently exchanged and exploited, causing generic decisions to be made. Moreover, services usually follow a naive, static, centralized approach where all exposed data is processed on central servers. This leads to increasing scalability and performance issues incurred by the massive amounts of data they need to process, which is especially challenging in an Internet of Things (IoT) environment dealing with high-volume and high-velocity data generated by various sensors. In addition, this also reduces local autonomy and data privacy of the set-up. Finally, existing end user tools that enable domain experts to create services and workflows do not scale in a non-entertainment or educational setting.

**Fig. 1:**
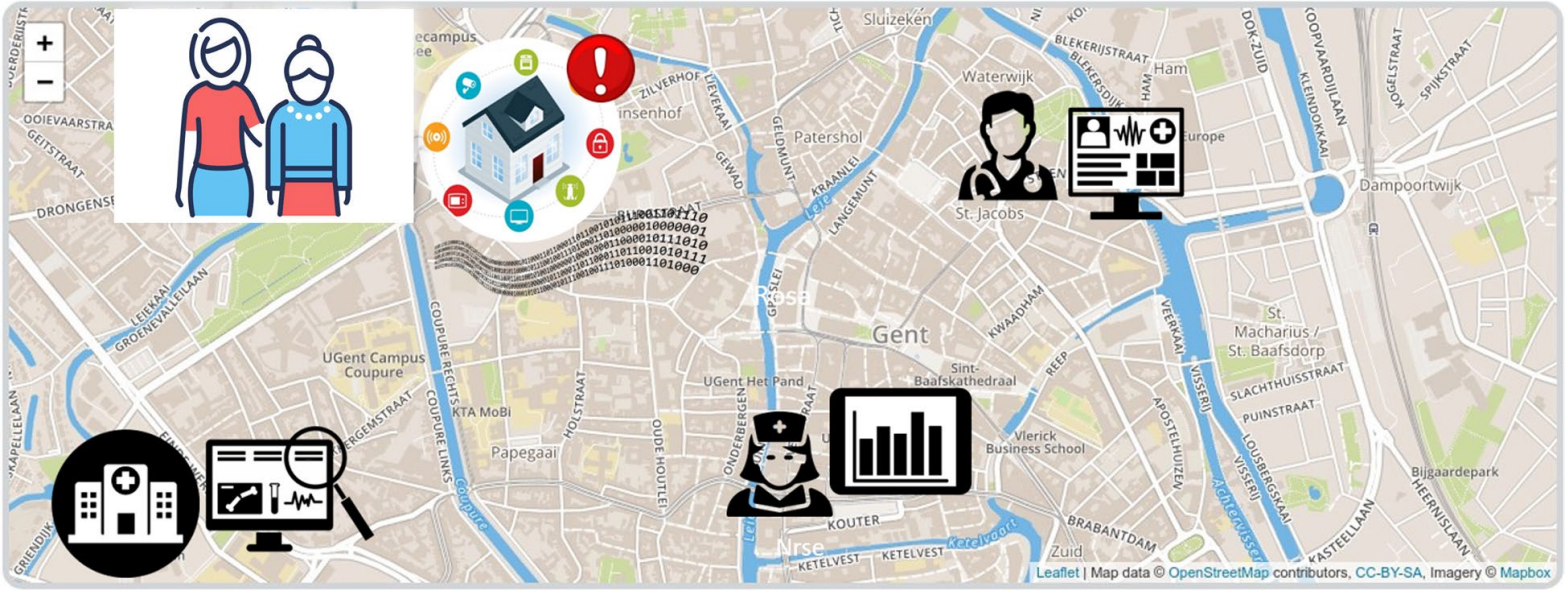
Visual overview of different possible caregivers that can be involved in following up homecare patients. These possible formal and informal caregivers can include a nurse, a general practitioner, hospital doctors, family members, etc. The data needed by these caregivers to optimally perform their care tasks and follow up the patient’s condition, is typically spread across the various involved stakeholders

Healthcare is an application domain in which these challenges are prevalent [5]. Homecare has become increasingly important over the last years, due to the gradual shift from acute to chronic care, where people are living longer with one or more chronic diseases, requiring more complex care [6]. The Belgian Healthcare Knowledge Centre (KCE) calculated in 2011 that a yearly increase between 1600 and 3500 beds in residential care centers and 46000 new employees are required in Flanders by 2025 to care for these elderly [6]. To reserve residential care for patients with more severe care needs, hospital stays are being shortened by making care delivery more transmural and enabling recovery at home and in service flats [7]. To reach the optimal scenario where only 1600 additional beds will be needed, KCE calculated that the accessibility of homecare should increase from 150000 patients to 225000 in Flanders to maintain a sustainable healthcare model [6]. To facilitate the shift to homecare, it is crucial to monitor and follow up the elderly at home in a dependable, accurate manner [8, 9]. Multiple formal and informal caregivers are involved in this process, as illustrated in Fig. 1. Typically, the patients and their service flats are equipped with multiple sensors to monitor patient and environment, and devices to steer home conditions such as its temperature and lights. Alarms are commonly generated by the services when anomalous situations are observed from the sensors, such as an abnormal blood pressure. Moreover, patients have a Personal Alarm System (PAS) to generate calls. A nurse regularly visits patients to handle the alarms and calls, and perform daily care and follow-up tasks. In addition, patients are followed up by their General Practitioner (GP), are registered as patients in their hospital, and often also have informal caregivers (e.g., a family member) to regularly check up on them. To perform their care tasks, all caregivers depend on the existing patient data and the data gathered by the different sources. However, these data are spread across the various involved stakeholders. As a result, it becomes challenging to deliver personalized homecare, for example when assigning the optimal caregiver to handle an alarm or call according to a patient’s profile [10]. Designing dynamic services that filter the data to detect alarming situations for which such an alarm or call should be generated, is specifically challenging within healthcare, as different diagnoses and contextual parameters will imply different conditions that need to be dynamically monitored. This requires custom services that are personalized, dynamic and performant in heterogeneous IoT environments. In addition, it is therefore also an existing challenge for these different stakeholders to organize themselves across organizations and leverage all available data optimally, in order to provide the best possible care for their patients [11].

Looking at these challenges from a technical perspective, four roles can be discerned: data providers, service providers, integrators and installers. Data providers expose the available data, on which service providers can build services used by integrators to compose workflows. Installers are people responsible for configuring all services, workflows and data provisioning tools to the needs of the patients and caregivers. For every role, the presented issues impose the following technical challenges:

- **Data providers:** How can they easily expose their data to other organizations in a reusable fashion, while making the explicit meaning of the data clear?
- **Service providers:** How can they step away from custom & non-reusable services, towards flexible & reusable services that can easily be constructed and configured based on the incoming data? How can they take all available background knowledge and contextual data of the patient profiles into account to provide personalized services? How can they intelligently deal with the high-volume and high-velocity data coming in and still offer services that meet the use case specific quality requirements such as performance, scalability, local autonomy and data privacy? How can they make the meaning of their services clear such that they can be easily picked up and reused by other organizations?
- **Integrators:** How can they move away from generic, static, manually constructed workflows towards flexible workflows, that can easily be configured and adapted based on the available services? How can they dynamically realize the desired functionality and adhere to use case specific quality constraints?
- **Installers:** How can they easily expose data and build services in a user-friendly way that minimizes the required effort and error probability of the configuration?

In addition, it is a technical challenge to create a closed feedback loop within an IoT platform that involves these different entities. To achieve this, the output of the service providers’ semantic services and the integrators’ workflows should be taken into account as feedback to update any local data sources as well as update how sensor data should be processed by the same or other services. This way, a closed loop would be created in the chain of how these different entities (data providers, services and workflows) are connected: data and knowledge would flow from the data providers to services and workflows, while knowledge and other feedback would flow back from the final services and/or workflows in the chain to the data providers and/or services in the beginning of the chain. This additional flowing back of feedback would create a loop and would thus close this chain.

### Semantic Web technologies

To resolve the individual challenges imposed to the different technical roles presented in the “Introduction” section, Semantic Web technologies can be leveraged. In general, data semantics allow a system to semantically annotate different heterogeneous data sources in a common, uniform, machine-interpretable format. In the context of IoT environments, this allows the integration of sensor data with various sources of domain knowledge, background knowledge and context information. By integrating all data, their meaning and context becomes clear, allowing personalized services to process the data and reason on it [12].

Semantic Web technologies are a set of recommended technologies that represent the vision of the World Wide Web Consortium (W3C) about applying data semantics to the web. At its core is the Resource Description Framework (RDF) [13], which is a graph-based model for expressing relationships between resources, utilizing subject-predicate-object triples to represent statements. Different formats exist to store RDF data, such as RDF/Turtle and N-Triples. Ontologies, which are often defined through the Web Ontology Language (OWL) [14], are semantic models that describe domain concepts, relationships and attributes in a formal way [12, 15]. The collection of RDF datasets on the web that are semantically interconnected is referred to as Linked Data [16]. Linked Data principles encourage the use of standardized Uniform Resource Identifiers (URIs) to interconnect datasets, promoting a web of interconnected and semantically enriched information. Using URIs improves the interoperability and linked nature of data on the Semantic Web. Triple stores, which essentially are databases specifically designed for storing RDF triples, play a crucial role in managing Semantic Web data. The SPARQL Protocol and RDF Query Language (SPARQL) [17] is another Semantic Web technology that is used to query and manipulate RDF data sources. Semantic reasoners such as RDFox [18] and VLog [19] can derive new implicit knowledge based on semantic descriptions and axioms defined in ontologies, by applying logical rules and inference techniques on these RDF triples. The computational complexity of semantic reasoning algorithms depends the expressivity of the underlying ontology [20], with various OWL sublanguages exhibiting distinct levels of expressiveness [21]. Stream reasoning is the research field that investigates the adoption of such semantic reasoning techniques for streaming data [22]. RDF Stream Processing (RSP) is a subdomain of stream reasoning that focuses on engines processing RDF data streams by continuously evaluating semantic queries on sliding or tumbling data windows that are continuously placed on top of the streams [23]. RSP-QL is a reference model unifying the semantics of different existing RSP approaches [24]. Cascading reasoning introduces multi-layered processing in stream reasoning with increasing reasoning expressivity across layers in order to balance the expressivity with the data velocity: low expressivity techniques in lower layers directly evaluate highly volatile data streams, selecting relevant portions for subsequent layers to process with increasing expressivity, resulting in the capability to perform highly expressive reasoning on reduced data streams [20].

Semantic Web technologies allow addressing the presented challenges in continuous homecare. From these challenges, it follows that there is a need to exchange, integrate, retrieve, manipulate and intelligently process the available healthcare data across different caregiver parties. To do this, machines need to understand the healthcare data in an unambiguous manner, in a similar way as humans do. Semantic Web technologies enable this and provide support for these various tasks to machines, which makes them a suitable solution [25]. In general, data semantics improve the interpretability, predictability and interoperability of a system [26], which is a big advantage, especially in healthcare [9]. In addition, Semantic Web technologies also allow building declarative solutions. This is an important requirement for solutions that tackle the challenges addressed in this paper: whenever you want to instrument certain processes or actions based on the healthcare data, you mainly want to declare in a general way *what* should happen (e.g., instructions to generate semantic data, queries, services, workflow steps), without already hard coding *how* this will happen [16]. Using Semantic Web technologies allows decoupling declarations from implementation.

### Paper objective, contribution and organization

The objective of this paper is to design a full semantic platform that allows solving the challenges imposed to the different technical roles presented in the “Introduction” section, and that introduces a closed feedback loop. To achieve this, the paper presents a generic, distributed, cascading reference architecture for such a platform, which consists of different semantic building blocks. Moreover, the paper presents a set of existing tools built on Semantic Web technologies that can be deployed as the different building blocks in an instantiation of the presented reference architecture. For every role, the presented architecture and building blocks should solve the imposed challenges in a performant manner. This translates the challenges into the following hypotheses:

- **Data providers:** By exposing data as Linked Data, their meaning and context becomes clear. This way, the data can easily be reused by different services.
- **Service providers:**
  (i) They can easily build data-driven, distributed semantic services upon the semantically exposed data sets by expressing their functionality as semantic definitions. These semantic definitions are represented by axioms and rules that define and extract a certain information need of the end user. A semantic reasoner can then derive new knowledge through definitions out of the incoming data, to obtain the requested information and possible corresponding actions from the data. As such, the functionality of each service is semantically clear.
  (ii) Additional personalized, local semantic services can be built that intelligently and efficiently filter the high-volume and high-velocity^1^ sensor data to only forward relevant data to the semantic reasoners according to medical domain knowledge, the patient profile, background knowledge and possible other context information.
- **Integrators:** By leveraging the semantic descriptions of services and other potential workflow steps, they can use reasoning to construct dynamic, easily configurable, cross-organizational semantic workflows that fulfill a particular functionality and that meet particular quality constraints.
- **Installers:** By using Semantic Web technologies, they can focus on creating the required semantic definitions, i.e., models, rules & axioms, without bothering with the technological and heterogeneous details of custom interfaces.

The contribution of this paper is the design of the reference architecture that allows validating the hypotheses and creates a closed feedback loop. Moreover, its contribution lies in the realization of such a semantic healthcare platform using various existing building blocks, that can be leveraged to optimize continuous homecare provisioning. This is proven through a demonstrator on a specific homecare use case scenario that focuses on personalized smart monitoring and cross-organizational treatment planning.

This paper is organized as follows. The “Methods” section presents the generic reference architecture and a selection of tools for the different architectural building blocks. The “Results” section presents the results of applying the reference architecture to the use case demonstrator, a performance evaluation of the different building blocks on this demonstrator, and a usability evaluation. Finally, the “Discussion” and “Conclusion” sections discuss and conclude how the platform allows solving the presented challenges, and validate the hypotheses.

## Methods

This section presents the reference architecture of the semantic healthcare platform, and a selection of its different building blocks to instantiate it.

### Reference architecture

**Fig. 2:**
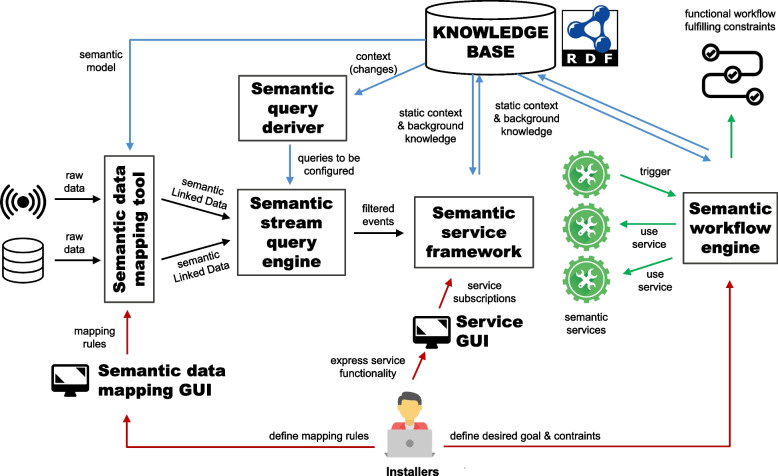
Reference architecture containing different building blocks built on Semantic Web technologies. This allows optimizing continuous homecare provisioning through distributed, data-driven semantic services and cross-organizational workflows

Figure 2 presents the reference architecture of the semantic healthcare platform that can be employed to deliver data-driven, personalized continuous homecare. It contains different semantic building blocks, for which various existing tools can be employed. In the remainder of this section, the architecture will be discussed in a tool-agnostic way.

To generate Linked Data from both static & streaming data sources, *semantic data mapping tools* can be used that make use of mapping languages. Installers should define the mapping rules to generate Linked Data through a *semantic data mapping Graphical User Interface (GUI)*. The generated rules are then forwarded to the mapping tool.

The semantic Linked Data generated by the *semantic data mapping tools* is forwarded to a *semantic stream query engine*. This is an RSP engine that continuously evaluates RSP queries on the data streams. These queries are derived and configured by the *semantic query deriver*. This component should be configured with a set of generic query templates that define for the given use case how to intelligently filter the relevant information from the incoming streams for the various services. By monitoring any changes to this data in the *Knowledge Base* and using these changes as a trigger to update the queries, this component ensures that the correct, contextually relevant semantic stream processing queries are evaluated at all times.

The queries configured by the *semantic query deriver* on the *semantic stream query engine* continuously filter the Linked Data delivered by the *semantic data mapping tool*. The filtered events are forwarded to *semantic service framework*. Through the service GUI provided for this framework, installers can configure the required services, by expressing their functionality as semantic queries and rules. The *semantic service framework* then performs the necessary semantic reasoning on the incoming filtered events to deliver the desired functionality. As such, including the given streaming data processing pipeline results in a cascading reasoning architecture.

The services can trigger the construction of a workflow in the *semantic workflow engine*, according to specified functionality and quality constraints. An example in homecare is a service that raises an alarm, which triggers a workflow to select a caregiver to handle this alarm. However, an installer can also express desired functionality by semantically specifying a goal and the constraints that should be met. This triggers the automatic, ad-hoc construction of a workflow by the engine.

Including the *semantic query deriver* into the architecture creates a closed feedback loop. Knowledge and actions generated through the semantic services or workflows can update the context in the *Knowledge Base*. This can trigger the *semantic query deriver* to update the context-aware queries on its turn, thereby closing the loop.

Note that instantiating the abstract building blocks in the reference architecture and configuring them is not a linear process. Installers should work closely together to ensure that the different building blocks in an instantiated platform are designed and configured in a compatible way. For example, the design of services in the *semantic service framework* through queries and rules implies certain input requirements of the data. These requirements should match the possible output format of the semantic queries configured by the *semantic query deriver* on the *semantic stream query engine*, since these services do not operate directly on the semantically annotated raw data.

### Building blocks

In the “Reference architecture” section, the presented reference architecture contains various building blocks. In this section, existing semantic tools are selected for these building blocks, to obtain an optimal instantiation of this architecture for a semantic healthcare platform. The overview is split up according to the different technical roles identified in the “Background” section.

#### Data providers: semantic exposure of high-velocity data

To generate Linked Data from heterogeneous data sources, different mapping languages exist [27]. These mapping languages can be considered as schema transformation descriptions, since they allow describing the mapping policy from source schema to target schema for the involved data sources. Existing mapping languages include the RDF Mapping Language (RML) [28], xR2RML [29], D2RML [30], Dataset Representation (D-REPR) [31], and more. In this paper, RML is chosen as it is considered the most popular mapping language to date [27]. RML is generic, declarative, and is defined as a superset of the RDB to RDF Mapping Language (R2RML), which is the W3C recommendation for mapping relational databases to RDF [32]. This way, the purpose of RML is to extend the applicability and broaden the scope of R2RML.

To perform the actual generation of RDF graphs from heterogeneous data sources with RML, different materialization implementations exist. Examples include RMLMapper [33], MapSDI [34], GeoTriples [35], and more [27]. RMLMapper is one of the first, well-known Java implementations to perform this task based on a set of defined declarative RML mapping rules [33]. Before the RDF generation starts, it sequentially ingests multiple data sources. This process loads all data in memory, imposing a strict limitation on the amount of data that can be ingested. Therefore, an alternative methodology was designed to parallelize the ingestion of data sources by distributing the ingestion over multiple nodes [36]. This way, the generation of RDF tasks is scaled with the volume of the data, allowing systems to generate RDF data in larger volumes. This methodology was implemented in Scala, resulting in the RMLStreamer materialization tool [36]. In this paper, the RMLStreamer tool is chosen as materialization implementation for the *semantic data mapping tool* in the architecture, since it is the only existing implementation using RML that supports the generation of RDF data from high-velocity streaming data [27].

#### Service providers: semantic service exposure on high-velocity data

Multiple tools allow providing semantic services on the generated high-velocity data. This section details two of them: DIVIDE and Streaming MASSIF.

##### DIVIDE

DIVIDE [37, 38] is a semantic IoT platform component that can be employed as the *semantic query deriver* in the architecture of Fig. 2. It can automatically derive queries for stream processing components in an adaptive and context-aware way. DIVIDE tries to solve the performance issues in existing IoT platforms for healthcare [25, 26, 39–41], which are caused by evaluating fixed, generic queries on high-velocity data streams. These platforms do this to avoid having to manually update the queries according to regularly changing application context, e.g., updating the monitored sensors when a patient’s diagnosis changes. DIVIDE avoids these performance issues by working with non-generic, sensor-specific queries for each RSP engine, allowing them to be continuously evaluated without the need to perform any more reasoning, thus improving query performance. It does this by performing upfront rule-based semantic reasoning on the current environmental context within the application, in order to automatically derive and configure the queries that filter observations that are relevant given the current context and the use case goal. To do so, it makes use of a new formalism that allows semantically representing generic query patterns in a declarative way. Through its design, DIVIDE can automatically adapt the configured queries upon context changes, ensuring they are contextually relevant at all times.

##### Streaming MASSIF

Streaming MASSIF [42] can be used as the *semantic service framework* in the reference architecture of Fig. 2. It is a cascading reasoning framework that enables to perform expressive semantic reasoning over high velocity streams. Streaming MASSIF is the first realization of the cascading reasoning vision. It employs three layers. The lowest layer is the selection layer, which efficiently selects those parts of the data stream that are relevant for further processing. In the reference architecture in Fig. 2, the selection layer is represented by the *semantic stream query engine*. This engine can be either C-Sprite, a reasoning system that employs an optimized reasoning algorithm for the efficient hierarchical reasoning on high-velocity data streams [43], or a regular RSP engine such as C-SPARQL [44], SPARQL_Stream_ [45], Yasper [46] and RSP4J [47]. The selections can then be abstracted in the abstraction layer, which allows defining high-level concepts and hide the low-level data details. These abstractions can then be used to define temporal dependencies in the temporal reasoning layer. All definitions can be easily provided in a declarative way. The layered approach of Streaming MASSIF allows services on top of these layers to easily define the data they are interested in. This can be seen as a very expressive publish/subscribe mechanism employing highly expressive reasoning to significantly decrease the subscription complexity. In this process, both temporal and standard logics can be incorporated to infer implicit data. Since Streaming MASSIF is the first concrete realization of the cascading reasoning vision and supports instrumenting concrete services through its multiple layers, it was chosen as a building block of the solution presented in this paper [42].

#### Integrators: functional semantic workflow engine

AMADEUS is an adaptive, goal-driven workflow composition and conflict-detection engine [48, 49]. In the reference architecture in Fig. 2, it can be employed as the *semantic workflow engine*. It solves the issues with common workflow planning systems [50–52], which can not provide personalized workflows, detect future conflicts between workflows, or have a limited notion of change [48]. Therefore, AMADEUS is state-aware: the workflow composition takes into account a semantic description of the current state or context. Thereby, it is driven by a Weighted State Transition logic: possible steps are declaratively described by the changes they will make to the state description, with a possible precondition. Hence, different step descriptions can be activated in different circumstances, for example to add additional steps to the plan, or to overrule other steps. AMADEUS composes a workflow that would bring the current state to the state described in the goal. To do this, it performs semantic reasoning on the semantic data, which include the state, steps and domain knowledge. The produced workflows adhere to the quality constraints set up by the given use case, which are captured in the step descriptions. The semantic state description reflects all what is known when the composition is performed and is iteratively modified by every step taken. Events produced by services, for example via Streaming MASSIF, can make external additions or alterations to the state description and trigger a recomposition to adhere to new constraints in a new workflow. In addition, AMADEUS is able to detect possible conflicts between different workflows, for instance between the current and newly adopted workflow. This conflict detection can be applied to find future issues when the current workflow is continued.

AMADEUS is implemented in the rule-based Notation3 (N3) Logic [53], which is a superset of RDF/Turtle. To compose the workflows, AMADEUS uses the EYE reasoner [54]. Applications can use AMADEUS through its Web API.

#### Installers: intuitive user interfaces

Multiple intuitive GUIs are available for installers. These include a GUI for Streaming MASSIF and GUIs to define RML mapping rules. In the architecture of Fig. 2, they can serve as the *service GUI* and *semantic data mapping GUI*, respectively.

##### Streaming MASSIF UI

**Fig. 3:**
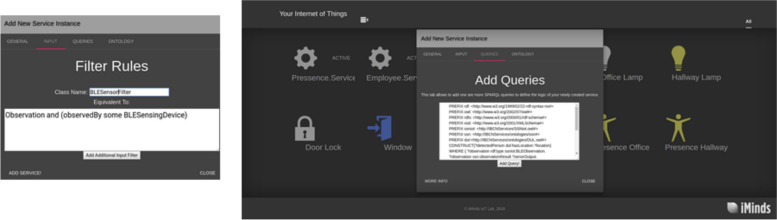
GUI to visually define service subscriptions in Streaming MASSIF [55]

To simplify the service subscription in Streaming MASSIF, a query language has been developed that unifies the various layers of Streaming MASSIF. Furthermore, as shown in Fig. 3, a GUI is provided to visually define these service subscriptions [55].

##### Graphical tools to define RML mapping rules

**Fig. 4:**
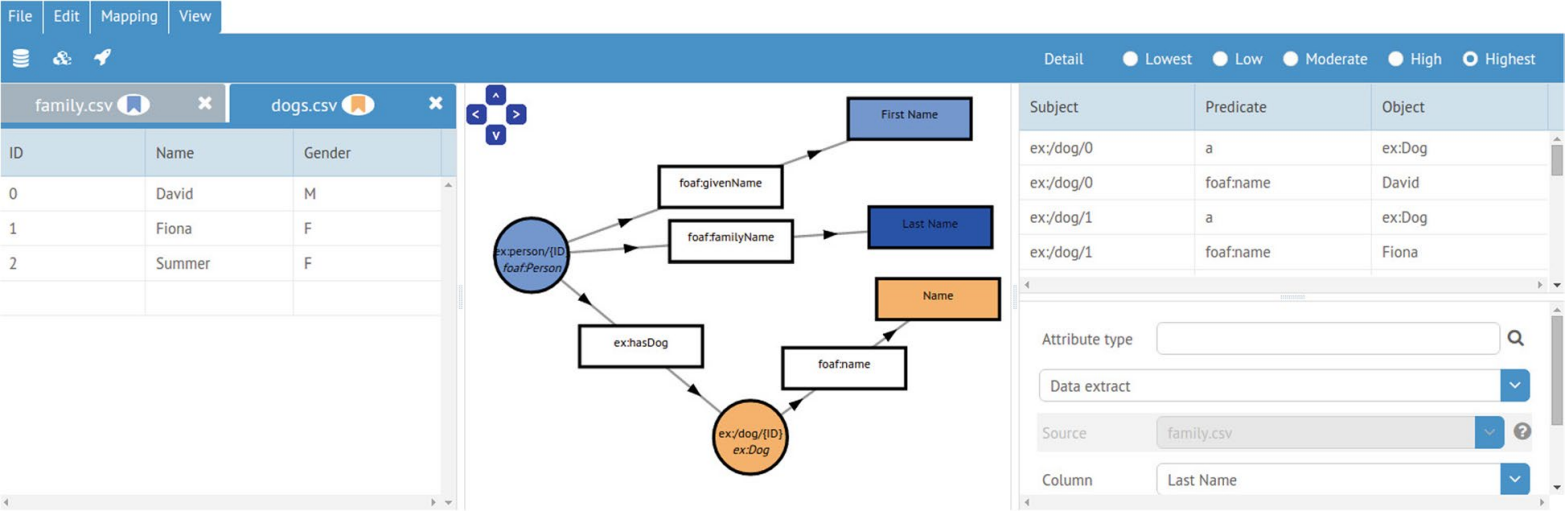
GUI of the RMLEditor [56]

The RMLEditor [56] is a graph-based visualization tool to facilitate the editing of RML mapping rules. Using the RMLEditor, installers can create and edit mapping rules, preview the RDF data that is generated from them, and export the rules. As such, it is always possible for installers with sufficient domain knowledge to generate Linked Data, even if they do not have knowledge about the Semantic Web or the used mapping language. The RMLEditor uses a visual notation for mapping rules called MapVOWL [57]. In its GUI, different ontologies and vocabularies can be used to define semantic annotations. The Linked Open Vocabularies (LOV) [58] are integrated to discover relevant classes, properties and datatypes. A screenshot of the RMLEditor’s GUI is shown in Fig. 4 [56].

Matey [59] is another tool that can be used to view and define Linked Data generation rules. It works with YARRRML [60], which is a human-readable, text-based representation language for RML mapping rules. YARRRML is a subset of the YAML data serialization language [61]. Matey works as a browser-based tool. It allows exporting the RML rules that correspond with the YARRRML representation. This way, installers can define RML mapping rules using the human-readable YARRRML representation, without requiring knowledge about the underlying mapping language. As such, Matey is more adequate for developers who are not Semantic Web experts, while the RMLEditor is more adequate for data owners who are not developers.

## Results

This section presents the results of applying the instantiated reference architecture, introduced in the “Methods” section, on a use case demonstrator in homecare. Moreover, it presents a performance evaluation of the different building blocks on this demonstrator, and a usability evaluation of the installer tools.

### Use case demonstrator

A demonstrator was built to showcase the feasibility of how the semantic healthcare platform based on the instantiated reference architecture can be used to optimize continuous homecare provisioning [62]. The demonstrator is implemented on a specific use case scenario in continuous homecare, focusing on personalized smart monitoring and the construction and cross-organizational coordination of patients’ treatment plans. This use case scenario has been designed in collaboration with different domain experts and companies involved in continuous homecare and hospital care. This way, it is ensured that this scenario is representative for a large set of real-world situations and problems within continuous homecare specifically and continuous healthcare in general. This will allow to generalize the performance and usability evaluation results in this paper towards the continuous homecare domain.

This section zooms in on the use case description, the demonstrator architecture, the scenario description and a web application of the demonstrator. An additional file zooms in on the technical implementation details of the Proof-of-Concept (PoC) implementation of the use case demonstrator [see Additional file 1].

#### Use case description

The demonstrator tells the story of Rosa, an elderly woman of 74 years old that lives in a service flat in Ghent, Belgium. To follow up on Rosa, her service flat is equipped with several environmental sensors measuring properties such as room temperature and humidity. Door sensors measure whether a door is currently open or closed. Moreover, Rosa has a wearable that continuously measures her steps, body temperature and heart rate. It also contains a PAS. Through multiple Bluetooth Low Energy (BLE) beacon sensors and a BLE tag integrated into her wearable, Rosa’s presence in the different rooms of the service flat can be detected.

Rosa’s medical profile contains the diagnosis of early stage dementia. Multiple people are part of her caregiver network. Nurse Suzy visits Rosa every afternoon, to assist with daily care. Dr. Wilson is Rosa’s GP. Rosa is also a known patient in a nearby hospital. Moreover, two informal caregivers of Rosa are registered: her daughter Holly, who works nearby and pays Rosa a daily visit around noon, and a neighbor Roger.

#### Demonstrator architecture

**Fig. 5:**
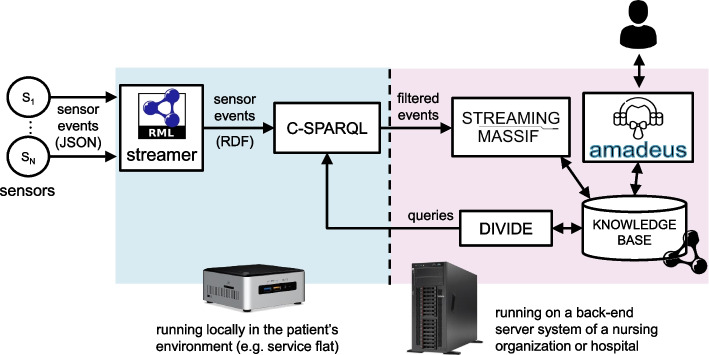
Architecture of the use case demonstrator

To monitor Rosa’s condition in real-time, the reference architecture in Fig. 2 is instantiated to the specific demonstrator architecture depicted in Fig. 5. The data processing pipeline consists of the RMLStreamer, C-SPARQL and Streaming MASSIF components. C-SPARQL was chosen as RSP engine as it is one of the most well-known existing RSP engines [22, 23]. Moreover, AMADEUS is deployed as semantic workflow engine. UI components are omitted from the demonstrator architecture.

The distributed architecture contains local and central components. RMLStreamer and C-SPARQL are local components that are deployed in the patient’s service flat, for example on an existing low-end local gateway device. They operate only for the patient living in that particular service flat. Streaming MASSIF, DIVIDE and AMADEUS run centrally on a back-end server in a server environment of either a nursing home or hospital. They perform their different tasks for all patients registered in the system.

In the smart monitoring pipeline, RMLStreamer maps the sensor observations from JSON syntax to RDF data. C-SPARQL filters the relevant RDF observations according to Rosa’s profile through queries derived by DIVIDE. In this use case, these queries are determined by the diseases Rosa is diagnosed with. Streaming MASSIF performs further abstraction and temporal reasoning to infer the severity and urgency of the events filtered by C-SPARQL. It implements a service that can detect alarming situations and generate notifications for them to the most appropriate person in the patient’s caregiver network. To decide this person, it takes into account the inferred event parameters and profile information such as already planned visits of caregivers.

AMADEUS is employed to compose semantic workflows representing possible treatment plans to a diagnosis in Rosa’s medical profile, and provide composed quality parameters for them to help the human doctor select the most optimal one. Quality constraints can be defined for the proposed plans on cost, probability of success, relapse risk, patient comfort, and such. The inputs of AMADEUS include the patient’s profile and medical domain knowledge about the options in the treatment of different diseases, which are defined by their input, output, functionality and quality parameters. AMADEUS’ automatic conflict detection between existing & new treatment plans can help a doctor in avoiding generating certain conflicts that they are unaware of.

#### Scenario description

To demonstrate how the building blocks of the demonstrator architecture work together in the presented use case, a specific scenario with multiple steps is designed.

##### Step 0 – Initial state

In its initial state, the smart monitoring pipeline is not yet activated. This means that no specific queries are evaluated on C-SPARQL. Instead, naive monitoring takes places where all sensor observations are forwarded to the central server.

##### Step 1 – Activating the smart monitoring pipeline

When the smart monitoring pipeline is activated, DIVIDE derives two personalized queries from the generic query patterns to be evaluated on C-SPARQL for Rosa.

The first query filters observations indicating that Rosa is longer than 30 minutes in her bathroom, without performing any movement. This query is derived because this might indicate that an accident has happened, e.g., Rosa has fallen. Because Rosa has dementia, there is a higher chance that she might forget to use her PAS in that case. This query monitors the bathroom’s BLE sensor and the wearable’s step detector.

The second query filters sensor observations which imply that Rosa has left her service flat. To detect this, it monitors the BLE sensor in the hallway and the main door’s sensor. Because Rosa has dementia, such events should be detected and notified to a caregiver, since being outside alone could possibly lead to a disorientation.

##### Step 2 – Colon cancer diagnosis

At a certain moment in time, Rosa’s medical profile is updated with the diagnosis of colon cancer by a medical specialist at the hospital, who examined Rosa after she complained to the nurse about pain in the stomach and intestines. This update automatically triggers DIVIDE to reevaluate the deployed C-SPARQL queries, without requiring any additional user intervention. As a result, one additional query is derived. It detects when Rosa’s body temperature exceeds 38^∘^C (38 degrees Celsius), i.e., when Rosa has a fever, by monitoring the sensor in Rosa’s wearable. This new query is derived because the medical domain knowledge states that complications or additional infections form a contraindication for several cancer treatments such as chemoradiotherapy, which means that continuing these treatments would be too dangerous [63]. Since fever might indicate an underlying infection, the medical domain knowledge therefore defines that cancer patients should be monitored for fever.

##### Step 3 – Constructing a treatment plan for colon cancer

To construct a treatment plan for Rosa’s colon cancer, AMADEUS is triggered by the hospital doctor. First, given Rosa’s profile, the defined treatments and their quality parameters, it composes two possible workflows: a plan consisting of neoadjuvant chemoradiotherapy followed by surgery, and a plan consisting of surgery only. The quality parameters for the presented plans include duration, cost, comfort, survival rate and relapse risk. Since the first plan has the highest survival rate and lowest relapse risk, it is selected by the doctor. This selection triggers AMADEUS to calculate a detailed workflow by adding timestamps to the different steps. In this case, the chemoradiotherapy step is split into four episodes in the hospital, with 30 days between each session. Every session can only be performed if there is no contraindication. After confirmation of the plan, it is added to Rosa’s current treatment plan.

##### Step 4 – Influenza infection yielding fever notifications

Five days before her next chemoradiotherapy session, Rosa gets infected with the influenza virus, causing her body temperature to start rising. Any body temperature observation exceeding the fever threshold of 38^∘^C is filtered by the deployed C-SPARQL query and sent to Streaming MASSIF.

The abstraction layer of Streaming MASSIF is configured to abstract the incoming sensor events according to several rules. A body temperature observation between 38.0^∘^C and 38.5^∘^C is a *low fever event*, one between 38.5^∘^C and 39.0^∘^C a *medium fever event*, and one above 39.0^∘^C a *high fever event*. Its temporal reasoning layer defines a *rising fever event* as a sequence of low, medium and high fever events within an hour.

Two queries are defined for the notification service instructed on top of Streaming MASSIF’s temporal reasoning layer. When a *low fever event* is detected, and a person in the patient’s caregiver network has already planned a visit to the patient on the current day, the first query notifies this person to check up on the patient during this visit. In that case, no other (medical) caregiver should be called. The second query notifies a medical caregiver from the patient’s caregiver network as quickly as possible when a *rising fever event* is detected.

In the use case scenario, in the morning of the given day, Rosa’s body temperature exceeds 38^∘^C. This event is filtered by C-SPARQL, and abstracted by Streaming MASSIF as a *low fever event*. The daily visit of Rosa’s daughter Holly around noon is still planned for the current day, causing Streaming MASSIF to generate a notification to Holly, indicating that she should check up on Rosa’s low fever during her planned visit. However, within an hour after the first low fever event, Rosa’s body temperature further rises to above 39^∘^C. Thus, Streaming MASSIF detects both a *medium fever event* and a *high fever event* in its abstraction layer, and thus a corresponding *rising fever event* in its temporal reasoning layer. Hence, the Streaming MASSIF service generates a notification to Rosa’s nurse Suzy to visit her with high priority.

##### Step 5 – Constructing a treatment plan for influenza

After Suzy’s examination, Rosa’s GP dr. Wilson is called and diagnoses her with the influenza virus, which is added to her medical profile. To construct a treatment plan for it, dr. Wilson can use AMADEUS. It proposes three possible plans: taking the oseltamivir medicine for ten days, taking the zanamivir medicine for eight days, or waiting for 16 days until the influenza goes over naturally. The durations of the plans resemble the expected time after which the influenza should be cured. Given Rosa’s situation, dr. Wilson decides to choose the first plan, which has the highest value for the comfort quality parameter. After selecting the plan, AMADEUS constructs the detailed workflow of taking the medication every day for a period of ten days.

##### Step 6 – Treatment plan conflict

AMADEUS performs a verification step to ensure that the newly added treatment plan, confirmed by dr. Wilson, does not yield any conflicts with Rosa’s current treatment plan. In this scenario, AMADEUS detects a conflict: Rosa’s next chemoradiotherapy session in the colon cancer treatment plan is scheduled in five days. However, since the influenza treatment plan still takes ten days, the influenza virus will not be cured by then, which forms a contraindication conflict. AMADEUS leaves resolving detected conflicts to its end users. In this case, dr. Wilson can do so by postponing the next chemoradiotherapy session until the influenza virus is fully cured.

#### Demonstrator web application

**Fig. 6:**
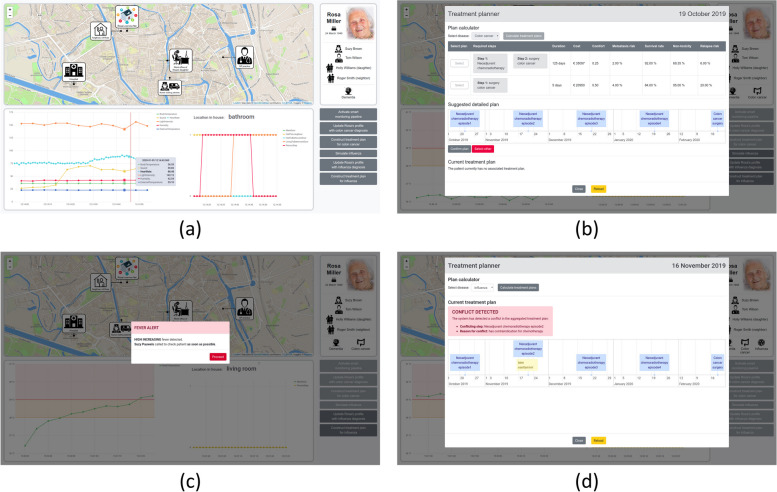
Screenshots of the web application built on top of the use case demonstrator’s PoC implementation. They correspond to different steps in the scenario: **a** Step 0 – Initial state; **b** Step 3 – Constructing a treatment plan for colon cancer; **c** Step 4 – Influenza infection yielding fever notifications; **d** Step 6 – Treatment plan conflict

To visually demonstrate the described use case scenario, a web application is designed [62] on top of a PoC implementation of the use case demonstrator [see Additional file 1]. It illustrates how medical care providers could follow up patients in homecare through the smart monitoring pipeline, in addition to the designed GUIs presented in the “Building blocks” section. More specifically, it contains multiple UI buttons to simulate the different steps of the scenario in the “Scenario description” section and shows a visualization throughout the simulation of Rosa’s profile, the location of Rosa and the people in her caregiver network, and the real-time observations generated by the sensors processed in the monitoring pipeline. Furthermore, it contains a UI to trigger AMADEUS and visualize its output. Figure 6 shows multiple screenshots of the web application corresponding to the different scenario steps. Moreover, a video of the demonstrator is available online at https://vimeo.com/380716692.

### Performance evaluation

This section evaluates the performance of the different building blocks in the architecture of the use case demonstrator presented in the “Use case demonstrator” section [62]. The main purpose of this evaluation is to evaluate the individual building blocks of the semantic healthcare platform on a single, fixed use case. For in-depth evaluations of the involved building blocks, we refer to the corresponding publications.

The evaluation is split up in three parts. The first part evaluates the data stream processing pipeline with RMLStreamer, C-SPARQL and Streaming MASSIF. Part two evaluates the DIVIDE query derivation. The third part evaluates AMADEUS.

For all evaluations, the local components in the demonstrator architecture in Fig. 5 are running on an Intel NUC, model D54250WYKH, which has a 1300 MHz dual-core Intel Core i5-4250U CPU (turbo frequency 2600 MHz) and 8 GB DDR3-1600 RAM. The central components are deployed on a virtual Ubuntu 18.04 server with a Intel Xeon E5620 2.40GHz CPU, and 12GB DDR3 1066 MHz RAM.

All evaluation results are aggregated in Table 1. For every evaluated component, the following subsections detail the evaluation cases, their rationales, the measured metrics, and how the measures were obtained to calculate the reported statistics.

#### Performance evaluation of the data stream processing pipeline

The evaluation of the data stream processing pipeline is performed separately for the three components. This approach is chosen because C-SPARQL performs continuous time-based processing of windows on the data streams, while RMLStreamer and Streaming MASSIF do event-based processing. Analyzing the components individually means that inherent networking delays are omitted.

##### RMLStreamer

For the RMLStreamer evaluation, the processing time is measured, which is defined as the difference between the time at which a JSON observation is sent on the TCP socket input stream of RMLStreamer, and the time at which the RDF observation arrives at the client consuming the TCP socket output stream. This client and the sensor simulator are both running on the same device as the RMLStreamer.

In Table 1, the RMLStreamer performance measures are reported for three different rates of incoming observations on the RMLStreamer: 1 observation per second, 7 observations per second and 14 observations per second. This maximum of 14 is chosen because the demonstrator contains 14 sensors. The reported numbers are aggregated over all observations generated during a simulation of 2 minutes.

**Table 1:** Results of the performance evaluation of the building blocks in the use case demonstrator’s architecture

Evaluated component	Measured metric	Evaluation case	Average value (ms)	Standard deviation (ms)
RMLStreamer	processing time	1 observation per second	8.1	3.1
		7 observations per second	11.8	7.1
		14 observations per second	13.5	8.3
C-SPARQL	query execution time	query filtering body temperature with 1 observation per second	12.2	3.3
		query filtering body temperature with 7 observations per second	15.2	9.1
		query filtering body temperature with 14 observations per second	26.4	23.5
Streaming MASSIF	processing time	fever event processing	1539.5	60.1
DIVIDE	processing time	query derivation	7249.5	175.8
AMADEUS	processing time	generating treatment plans for colon cancer	190.8	1.4
		generating treatment plans for influenza	88.6	1.7
		aggregating treatment plans and performing conflict detection	1335.7	3.8

##### C-SPARQL

For the C-SPARQL evaluation, the execution time is measured of the query that is filtering Rosa’s body temperature after she is diagnosed with colon cancer. This is the only query that is important for the scenario of the demonstrator, since the other two deployed queries never filter any event during the scenario.

Table 1 reports the evaluation results for the three rates of incoming RDF observations. For C-SPARQL, this defines the number of observations in the data window on which the queries are evaluated. For every rate, exactly one body temperature observation higher than 38^∘^C is generated per second. Hence, this resembles the period in the scenario when Rosa has a fever. Thus, the reported measures are for query executions that each yield exactly one result, being the most recent high body temperature observation. The query is evaluated every 3 seconds on a 5-second window. The reported numbers are aggregated over all query executions during a 2-minute simulation.

Note that the evaluation results report measures about the query execution times, and not the processing times of an observation. This is because the C-SPARQL query evaluation is not event-based but a continuous, periodic process. The total processing time per observation consists of the waiting time before the window trigger and query evaluation, and the query execution time. The waiting time is upper bounded by the time period between consecutive query evaluations, which is 3 seconds in the demonstrator. Since the actual waiting time is inherent to the system, depends on the mutual initialization of components, and is not dependent on the query bodies and data models, it is not included in the reported results.

##### Streaming MASSIF

For the evaluation of Streaming MASSIF, the processing time of an incoming event is measured. This is defined as the difference between the event’s arrival time and the time at which the notification (to either Rosa’s daughter or nurse) leaves the system. The reported numbers in the results in Table 1 are aggregated over all processed events in a simulation of 3 minutes, where Rosa’s body temperature is gradually increased from 38.3^∘^C up to 39.1^∘^C. The period between incoming events in Streaming MASSIF is equal to the output rate of the evaluated C-SPARQL query, which is 3 seconds.

#### Performance evaluation of DIVIDE

The evaluation of DIVIDE measures the processing time of the query derivation on the context associated to Rosa, which includes both the dementia and colon cancer diagnoses. DIVIDE performs the semantic reasoning during the query derivation in three parallel threads, where each thread is responsible for deriving the RSP queries from one of the generic query templates. The output consists of the three queries described in the demonstrator’s scenario. The processing time is measured from start to completion of the parallel reasoning processes. All networking overhead for registering the context to DIVIDE, which triggers the query derivation, and registering the resulting queries on C-SPARQL, is not included in the results reported in Table 1. These are aggregated over 30 runs, excluding 3 warm-up and 2 cool-down runs.

#### Performance evaluation of AMADEUS

For the evaluation of AMADEUS, the processing times are measured of a request to the AMADEUS Web API for the three most important cases in the demonstrator’s scenario: (1) requesting possible treatment plans for colon cancer, (2) requesting possible treatment plans for influenza, and (3) adding the chosen influenza treatment plan to the existing treatment plan for colon cancer, including the conflict detection. The processing time corresponds to the response time of the AMADEUS Web API, which mainly represents the duration of the started EYE reasoner process. The results in Table 1 are measured over 30 runs, excluding 3 warm-up and 2 cool-down runs.

### Usability evaluation

This section discusses the usability evaluations of the installer tools in the semantic healthcare platform: the RMLEditor and Streaming MASSIF with its GUI. The evaluations make use of the System Usability Scale (SUS), which is a well-known, rapid method for gathering usability ratings for a technology through a questionnaire [64]. It is known for being concise, applicable across various technologies, and effective in scenarios with limited sample sizes [65]. The SUS measures the user satisfaction of a technology, but not its effectiveness or the efficiency, which is an important consideration when interpreting SUS-scores. The usability of DIVIDE and AMADEUS has not been evaluated.

#### Usability evaluation of RMLEditor and MapVOWL

In a previous publication, Heyvaert et al. have evaluated the usability of the RMLEditor and its visual mapping rule notation MapVOWL [57]. To this end, they first evaluated whether the MapVOWL graph-based representation of RML mapping rules has a higher human processing accuracy and preference than the classic textual representation of RML rules. Based on the evaluation, the authors concluded that users with knowledge of RML exhibit no difference in accuracy of processing the representations, but do have a preference for MapVOWL to visualize and edit rules. As a second step, the authors evaluated the usability of the graph-based RMLEditor to the RMLx Visual Editor, which is form-based editor to show and edit RML mapping rules [66, 67]. This evaluation showed no difference in the accuracy of creating mapping rules between both editors, but revealed a higher user satisfaction of the RMLEditor through a SUS-score of 82.75 compared to 42 for the RMLx Visual Editor. This is mainly caused by the usage of MapVOWL. For details about the evaluation set-up and results of both evaluations, we refer to the original publication of Heyvaert et al. [57].

#### Usability evaluation of Streaming MASSIF

To evaluate the usability of using and configuring semantic services with Streaming MASSIF for installers, it is compared with Kafka Streams. This is a stream processing library provided by Apache Kafka that enables developers to build scalable and fault-tolerant real-time applications and microservices [68]. It allows Kafka consumers to process continuous streams of data records from Kafka topics, supporting tasks such as data transformation, aggregation, and event-driven computations.

##### Evaluation set-up

The evaluation was configured through an online questionnaire. Potential participants were approached from imec and Ghent University. Those agreeing to participate were provided with a short introduction of Streaming MASSIF and Kafka Streams to read through. After the introduction, the actual evaluation was performed together with one of the researchers, constituting of four consecutive parts.

**Part 1.** In this part of the evaluation, the questionnaire queried the participant’s socio-demographics. Moreover, questions were asked about their experience & familiarity with Linked Data & Semantic Web technologies, stream processing in general, Streaming MASSIF, and Kafka.

**Part 2.** The second part of the evaluation presented a short text about the fictional evaluation use case, which was the use case of the demonstrator presented in the “Use case demonstrator” section. It described the task that should be performed by the participant, which was about step 4 of the described scenario, i.e., the configuration of a service filtering the alarming situation of a rising fever event for patient Rosa with the influenza diagnosis. More specifically, this text reads as follows: “*In the patient room of the future, rooms are equipped with many sensors that capture their environment. These sensors allow to monitor both patients and the status of the room. Let’s consider the presence of a light, sound, and body temperature sensor present in the room. To process this data in a meaningful way, this data needs to be combined with background knowledge regarding the hospital and the patient that is being treated in the room. In this use case, we want to monitor to see if our patients are not being exposed to any alarming situation. For example, let’s assume our patient has influenza. The background knowledge about our patient will then describe that our patient with influenza should be monitored for body temperature values. Therefore we should monitor the body temperature sensors in the room, in order to detect alarming situations. In this case, an alarming situation occurs when the body temperature of our patient is rising too quickly in a limited time span.*”

After reading this text and following a short tutorial for both Streaming MASSIF and Kafka Streams, the participants were requested to complete this task with both tools. To have a variation in which tool was used first for the task, the participants were randomly assigned in two groups of equal size. The correctness of performing the task with both tools was assessed by the present researcher.

**Part 3.** The third part of the evaluation consisted of a multiple-choice questionnaire assessing how well the participants understand Streaming MASSIF. The questions asked and their corresponding correct answer(s) were the following:

1. How does one filter data in Streaming MASSIF? Correct answers: by defining a filter, by abstracting the data, by defining a Complex Event Processing pattern
2. How does one enrich events from the data stream in Streaming MASSIF? Correct answers: by defining a CONSTRUCT query in a filter, by abstracting the data through reasoning
3. How does one abstract the data in Streaming MASSIF? Correct answer: by defining ontological patterns
4. How does one detect temporal dependencies in Streaming MASSIF? Correct answer: by defining a temporal pattern

**Part 4.** The final part was a post-assessment questioning the usability of both tools. First, the difficulty of performing the use case task with the tools was rated on a 7-point Likert scale from extremely difficult to extremely easy. Similarly, confidence in successful completion was rated on a 7-point Likert scale from strongly agree to strongly disagree. Second, the SUS-score was obtained for both tools. Third, the overall user-friendliness of the tools was rated on a 7-point Likert scale from awful to excellent.

##### Evaluation results

**Part 1.** Eight participants were recruited. They were all male researchers and students from imec and Ghent University between 22 and 28 years old, all but one holding a master’s degree. One participant considered himself a novice on Linked Data and Semantic Web technologies, five were developing knowledge in this domain, one was proficient, and one was an expert. Concerning stream processing in general, four participants were developing knowledge and four were novices. Six participants had already heard of both Streaming MASSIF and Kafka, of which two had already used the latter. The other two participants had never heard of both.

**Part 2.** All participants successfully completed the task on the presented use case with both evaluated tools.

**Part 3.** The questions about Streaming MASSIF were answered by all participants. For question 1, one participant selected all three correct answers. Two participants selected two of them, and the other five selected one correct answer. For question 2, three participants selected both correct answers. The other five participants selected one correct answer. For question 3, five participants selected the single correct answer. Three people also selected an additional wrong answer. For question 4, the same happened for two participants, while all of them selected the correct answer.

**Part 4.** The difficulty of performing the use case task was rated higher for Kafka Streams compared to Streaming MASSIF by seven participants. Five participants rated completing the task with Streaming MASSIF moderately or slightly easy, compared to only two for Kafka Streams. Moreover, regarding confidence in successful completion of the tasks, two participants revealed a higher confidence when using Streaming MASSIF, while the others revealed equal confidence. This confidence was rated positively (slightly agree, agree or strongly agree) by seven participants for Streaming MASSIF. In addition, the average obtained SUS-score of Streaming MASSIF was 72.81, compared to 53.75 for Kafka Streams. Inspecting the individual SUS-scores, they were higher for Streaming MASSIF for six participants, while both scores were equal for the other two participants. Finally, the overall user-friendliness of Streaming MASSIF was rated higher than that of Kafka Streams by five participants, while the other three participants rated it equally well. All participants rated the overall user-friendliness of Streaming MASSIF as good or excellent, compared to a rating of good or OK for Kafka Streams by six participants.

## Discussion

This section discusses how the presented existing building blocks built upon Semantic Web technologies (“Building blocks” section) can help solving the challenges related to every individual role in the instantiated reference architecture (“Reference architecture” section). To do so, relevant insights from designing the use case demonstrator (“Use case demonstrator” section) and evaluating our PoC implementation of this demonstrator (“Performance evaluation” and “Usability evaluation” sections) are shared as well. This way, the hypotheses presented in the “Paper objective, contribution and organization” section are validated. This validation is justified by the fact that the use case scenario of the demonstrator use case is representative to the real world within the continuous homecare domain. This domain is an actual, currently relevant application domain within healthcare, due to the shift to homecare, which is demonstrated in Flanders by the analysis and calculations made by the KCE.

### Data providers

Semantic Web technologies offer the tools to data providers to formally describe different heterogeneous data sources in a uniform, common, machine-interpretable format. This way, reusing data sources defined as Linked Data across organizations and applications becomes possible. Exposing data from various sources as Linked Data is possible through RML mappings. RMLMapper is a tool that can process such mapping rules and generate Linked Data. RMLStreamer is another tool that parallelizes the Linked Data generation process as much as possible. This reduces its memory footprint and thus allows efficiently generating Linked Data in streaming use cases as well [36].

The evaluation results with the use case demonstrator prove that RMLStreamer can efficiently process and map JSON observations to RDF data. For a scenario where 14 observations per second are generated, the average processing time is only 13.5 ms.

To summarize, hypothesis (a) of this paper can be validated by following the Linked Data approach and using technologies and tools such as RML and RMLStreamer.

### Service providers

Service providers are responsible to build services upon the data exposed by the data providers. Different semantic building blocks such as Streaming MASSIF and DIVIDE in combination with engines such as C-Sprite or C-SPARQL allow moving away from the manual configuration and non-reusable services.

Both Streaming MASSIF and DIVIDE take the available background knowledge and contextual data of the patient profiles into account when performing semantic reasoning. This way, they allow designing personalized services. Moreover, they are both designed to deal with high-volume and high-velocity data streams in many healthcare use cases. They are designed for a distributed, cascading reasoning architecture, where some data stream processing is already performed in the edge of the IoT network, for example on a device in the local environment of the patient. This is done in the selection layer of Streaming MASSIF, where different engines such as C-Sprite, C-SPARQL or another regular RSP engine can be employed. C-Sprite is especially useful when efficient reasoning needs to be performed with many hierarchical concepts.

DIVIDE is responsible for configuring the queries that are evaluated on the local RSP engine. Through the defined generic query templates, it performs semantic reasoning to derive the specific RSP queries that are relevant with the given environmental context, every time this context (e.g., the medical profile) changes. The evaluation results show that the query derivation for the use case demonstrator takes a little over 7 seconds. This is relatively high, however, it is only performed upon context changes, of which the frequency is a few orders of magnitudes smaller than the frequency of the RSP query evaluation. DIVIDE ensures that only the relevant data is filtered, and that no real-time reasoning is required during the query evaluation. Hence, this query evaluation is very efficient. This is shown in the C-SPARQL evaluation results, which report an average query execution time of only 26.4 ms for a data stream containing 14 RDF observations per second. It should be noted that the query evaluation is also performant on low-end devices with few resources, which often occur in the IoT edge, even for data streams with a higher data velocity [37]. This allows for improved system performance, scalability, and local autonomy.

Moreover, by embedding DIVIDE into the reference architecture, privacy by design is enabled to some extent [69]. Multiple foundational principles of privacy by design are partly addressed this way, such as visibility & transparency, user-centric design and proactiveness, helping installers to design applications with the user data privacy in mind. Thus, this leaves an additional responsibility to installers, which implies the need for proper education and supporting GUIs for user-centric privacy management. Nevertheless, the use of DIVIDE in the distributed reference architecture system of course does not guarantee privacy as such. To achieve optimal privacy, it should be combined with additional privacy measures, both generic (e.g., strong cryptography) and use case specific measures. To this end, much existing research can be consulted [70]. Moreover, privacy measures should also consider other aspects than the transmission of data, such as privacy of data stored on devices.

In its abstraction and temporal reasoning layer, Streaming MASSIF allows defining functionality through new semantic axioms and rules in a relatively simple way [see Additional file 1]. Similarly for the services instructed on top of these layers, simple queries can be declared to describe the functionality of the service. This allows them to be reused in a user-friendly way. Streaming MASSIF also delivers performant semantic services, as is shown through the evaluation results on the use case demonstrator. On average, it takes a little over 1.5 seconds to generate the correct notification corresponding to a fever event received from the selection layer. Considering this processing includes expressive semantic reasoning on the full ontology with all medical domain knowledge and Rosa’s profile information, this is a performant result.

Based on this discussion, it can be concluded that hypothesis (b) of this paper can be validated by using the DIVIDE and Streaming MASSIF building blocks in a distributed, cascading reasoning architecture. More specifically, Streaming MASSIF validates sub-hypothesis (i), while using DIVIDE allows validating sub-hypothesis (ii).

### Integrators

Integrators compose workflows that fulfill a particular functionality. AMADEUS uses semantics to move away from generic, static, manually constructed workflows that cannot easily be coordinated across organizations. It performs semantic reasoning to compose possible workflows using the semantic description of all context and profile information, possible workflow steps, functionality of services, and quality parameters offered by them. This way, the resulting workflow always offers the desired functionality and meets quality constraints dynamically chosen by the end user.

To make this more tangible, the use case demonstrator focuses on an example where workflows represent medical treatment plans for a disease. In this example, potential steps in the treatment of diseases represent possible workflow steps. The semantic description of a step defines when applying this step is useful (e.g., for which diseases, given which preconditions), what the impact on the state and context is (e.g., how much does it cure the patient’s disease), what the quality parameters of this step are (e.g., what is the patient comfort or the treatment cost), and what possible contraindications exist for this step (e.g., what other diagnoses cannot be present to take this step). This makes it possible to create personalized, dynamic treatment plans that take into account particular quality constraints about the treatment.

An additional advantage of using AMADEUS is its ability to perform automatic conflict detection between workflows. In the use case demonstrator, a possible conflict is a contraindication for a new treatment plan. This detection is particularly interesting in cross-organizational environments, like in the use case scenario. The original colon cancer treatment plan was constructed by a hospital doctor, while the new influenza treatment plan was created by Rosa’s GP. Hence, this demonstrates how AMADEUS can help improving the communication and coordination of workflows across the different organizations and stakeholders involved in Rosa’s caregiving.

The evaluation results of AMADEUS on the demonstrator show that it can efficiently generate its dynamic workflows. All possible treatment plans for both the colon cancer and influenza diagnoses are generated in less than 200 ms on average. The conflict detection takes on average a little above 1.3 s, which is still acceptable given the fact that AMADEUS should not be deployed in a real-time data processing pipeline.

To summarize, it can be concluded that the design and performance of AMADEUS allows validating hypothesis (c) of this paper.

### Installers

Installers are people responsible for configuring all data provisioning tools, services, and workflows. For the first two aspects, GUIs for the semantic tools are available.

Defining RML mapping rules for the Linked Data generation is a tedious and time-consuming work, as mappings need to be created for each type of input data source to the semantic ontology model. To make this process much easier, either the RMLEditor or Matey can be used. These tools have an optimized GUI to easily generate, visualize and export mapping rules and/or Linked Data. Matey is most suited for developers who do not have knowledge about Semantic Web technologies, while the RMLEditor is most useful for data owners who are no developers. Specifically focusing on RMLEditor, previous research has shown that using the RMLEditor and its graph-based MapVOWL representation has a high usability compared to alternatives [57].

In addition, a GUI allows installers to declare the axioms, rules and queries that define the services in Streaming MASSIF, without having to bother with the underlying technological details. However, additional research is still needed to design a GUI to properly configure DIVIDE and its generic query templates. The usability evaluation of Streaming MASSIF in the “Usability evaluation of Streaming MASSIF” section proves that configuring semantic services with Streaming MASSIF is considered relatively easy and user-friendly by almost all participants. The average SUS-score of 72.81 of Streaming MASSIF translates to a usability rating of *good* [65]. With a proper introduction and a small tutorial, users can confidently and successfully use Streaming MASSIF and mostly correctly assess how it works. Altogether, these are positive results.

To conclude, the available GUIs validate hypothesis (d) of this paper.

In addition, it should be noted that the usability of two other building blocks, DIVIDE and AMADEUS, has not been formally evaluated within the scope of this paper. Therefore, it is important to zoom in on the required configuration workload of these tools for installers.

Looking at DIVIDE, its configuration workload mainly consists of defining the generic query templates. To do so, it should be noted that the DIVIDE implementation includes a parser that can automatically translate SPARQL queries into the required internal representation format of generic query templates used by DIVIDE [37]. This way, end users can configure DIVIDE in a straightforward way, without having to know its internal representation format. To define these SPARQL queries, existing SPARQL query configuration UI tools could possibly be employed as well, to further reduce the configuration workload. During its runtime, no manual (re)configuration of DIVIDE is required, as its internal algorithm will automatically ensure that the correct stream processing queries are deployed at all times. In addition, the configuration of DIVIDE also includes defining a set of properties that can overwrite its default values, which is easily possible through properties files. To make this configuration more easy, a simple UI tool could be designed on top of these properties files. Thus, altogether, the configuration workload of DIVIDE is relatively small compared to the configuration workload of the other components. This workload is significantly higher for configuring the RML mappings for RMLStreamer, and will typically become more extensive for the rules and queries for the different layers of Streaming MASSIF as well.

Zooming in on AMADEUS, it should be noted that the biggest part of the AMADEUS configuration actually represents medical domain knowledge, and should thus be captured in the used ontology. This includes the definition of policies, all possible workflow step descriptions, and additionally relevant medical domain knowledge such as preconditions of certain steps or contraindications. This knowledge should thus be captured from the involved domain experts in the ontology design process, for which existing ontology editors such as PoolParty or Topbraid Composer can be used [71, 72]. This configuration should only be done once for a given use case domain. In addition, the state description used by AMADEUS is captured through the current use case context in the knowledge base. Hence, the only use case specific configuration element is the goal of the workflow generation. This configuration is limited to a single rule for a given type of workflow, implying a small configuration workload for AMADEUS. In terms of end user interaction after the workflow generation process, an end user is required to select one of the proposed workflows to add it to the user’s set of workflows in the knowledge base and trigger the conflict detection. The design of the web application involves a use case specific UI element that specifically focuses on this selection and the presentation of possible workflows and conflicts (Fig. 6b and d). This part of the web application could serve as a starting point to design a generic UI tool for AMADEUS.

## Conclusion

The impact and contribution of this paper is the design of a reference architecture for a semantic healthcare platform that can be leveraged to optimize continuous homecare provisioning use cases. To this end, the distributed, cascading reasoning architecture is instantiated with different existing building blocks, built upon Semantic Web technologies. This architecture allows solving the challenges associated to the different roles involved in continuous care solutions. For data providers, the architecture allows exposing data as Linked Data to services and other organizations in reusable fashion, using declarative mapping rules. This Linked Data can be efficiently generated in use cases dealing with high-velocity streaming data. Concerning service providers, the architecture allows designing dynamic, use case specific, data-driven, personalized, reusable services. These services are defined by declaratively expressing their functionality and meaning as semantic definitions, and operate on the data abstractions and insights generated by stream reasoning queries. These queries efficiently process the generated Linked Data in a cascading reasoning pipeline, which allows for improved system performance, scalability, local autonomy, and data privacy to a certain extent. Moreover, considering service integrators, the architecture allows constructing dynamic workflows of different services or specific functionality described through declarative semantic descriptions. Conflicts can be automatically detected between constructed workflows, improving their coordination across organizations and stakeholders involved in the care provisioning of patients. By chaining all building blocks, a closed feedback loop is created: knowledge generated through services and workflows can result in context changes, which are automatically reflected in the adaptive, context-aware stream reasoning queries. Finally, for installers, different GUIs are available to easily expose Linked Data and build dynamic services in a user-friendly way. This allows installers to configure the system without requiring knowledge about technical details, minimizing the manual effort and risk of configuration errors. Through the performance evaluation on a PoC implementation of a realistic use case demonstrator in homecare, the paper has also shown that the platform can be successfully realized, and that the different building blocks of the instantiated reference architecture can perform their tasks in an efficient way.

Future work could involve multiple different pathways. First, since the solutions presented in this paper are targeted towards continuous homecare provisioning, a real-world production environment of the presented semantic healthcare platform should further address the involved privacy and security requirements. More specifically, for privacy, it is necessary to implement a set of additional privacy measures. These may include traditional methods like robust cryptography and access control mechanisms. In addition, other privacy solutions should be tailored to meet specific requirements of the use case. Second, to improve the overall usability of the presented semantic healthcare platform for the installers of the system, additional measures should be implemented. More specifically, UI tools should be designed for DIVIDE to more easily configure DIVIDE and its different generic query templates. In addition, the part of the web application built to configure AMADEUS for the demonstrator use case could be further generalized towards a generic UI tool for AMADEUS. The usability of these new and existing UI tools involved in the platform should be further evaluated as well, including their effectiveness and efficiency. Third, individual improvements of the different building blocks in the presented reference architecture, such as DIVIDE, Streaming MASSIF and AMADEUS, could further extend their applicability to different use cases and the available support for specific types of services and workflows. For concrete pointers on how to improve them, we refer to the specific publications about these individual building blocks. Fourth, future work should include the application and validation of the presented reference architecture on other healthcare use cases that exhibit requirements similar to homecare, such as system performance, local autonomy, data privacy, automation, dealing with dynamic environments, and cross-organizational workflows. Moreover, it might as well be interesting to investigate its generalization towards other IoT applications domains, such as smart cities or smart home and automation. This would allow to further evaluate the platform’s performance and usability on other use cases as well.

### Supplementary Information

**Additional file 1.** Implementation details of the PoC implementation of the use case demonstrator presented in the “Use case demonstrator” section. This file contains descriptions and listings about the implementation and configuration of RMLStreamer, DIVIDE, C-SPARQL, Streaming MASSIF and AMADEUS.

## Data Availability

Code and set-up documentation of the different tools are available online. For DIVIDE, this information is available at https://github.com/IBCNServices/DIVIDE. For Streaming MASSIF, this is provided at https://github.com/IBCNServices/StreamingMASSIF. For AMADEUS, this is available at https://github.com/IDLabResearch/AMADEUS-workflows. For the RMLStreamer and RMLMapper, more information is available at https://github.com/RMLio/RMLStreamer and https://github.com/RMLio/rmlmapper-java, respectively. Similarly, https://rml.io/tools/rmleditor/ and https://w3id.org/yarrrml/matey/ contain extra information about the RMLEditor and Matey, respectively. For C-Sprite, extra info is available at https://github.com/IBCNServices/C-Sprite. The ACCIO ontology used in the use case demonstrator is available online at https://github.com/IBCNServices/Accio-Ontology/tree/gh-pages.

## Abbreviations

ACCIO: Ambient-aware provisioning of Continuous Care for Intramural Organizations
API: Application Programming Interface
BLE: Bluetooth Low Energy
C-SPARQL: Continuous SPARQL
CPU: Central Processing Unit
DDR: Double Data Rate
e.g.: exempli gratia – for example
FWO: Research Foundation – Flanders (Dutch: *Fonds Wetenschappelijk Onderzoek – Vlaanderen*)
GP: General Practitioner
GUI: Graphical User Interface
i.e.: id est – that is (in other words)
IoT: Internet of Things
JSON: JavaScript Object Notation
LOV: Linked Open Vocabularies
N3: Notation3
OWL: Web Ontology Language
PAS: Personal Alarm System
PoC: Proof-of-Concept
R2RML: RDB to RDF Mapping Language
RAM: Random Access Memory
RDF: Resource Description Framework
RML: RDF Mapping Language
RSP: RDF Stream Processing
SNOMED CT: Systematized Nomenclature of Medicine Clinical Terms
SOA: Service Oriented Architecture
SPARQL: SPARQL Protocol And RDF Query Language
Turtle: Terse RDF Triple Language
UI: User Interface
W3C: World Wide Web Consortium
YAML: YAML Ain’t Markup Language

## Footnotes

1 In this paper, we define *high velocity data* as streaming data where the total rate of observations is at least twice as high as the evaluation frequency of the involved queries. This implies window-based processing will have to deal with multiple events per query evaluation, in comparison to event-based processing. We exclude streaming use cases with extremely high velocities that need to deal with strict (hard) real-time requirements.
