# Supplementary material for: Optimized continuous homecare provisioning through distributed data-driven semantic services and cross-organizational workflows
Source: J Biomed Semantics. 2024 Jun 6;15:9. doi: 10.1186/s13326-024-00303-4 (PMC11154993; doi:10.1186/s13326-024-00303-4)
Supplement: Supplementary file 1 — Additional file 1. Implementation details of the PoC implementation of the use case demonstrator presented in the “Use case demonstrator” section. This file contains descriptions and listings about the implementation and configuration of RMLStreamer, DIVIDE, C-SPARQL, Streaming MASSIF and AMADEUS. [file 13326_2024_303_MOESM1_ESM.pdf]

Implementation Details of the Use Case Demonstrator

Mathias De Brouwer, Pieter Bonte, Dörthe Arndt,
Miel Vander Sande, Anastasia Dimou, Ruben Verborgh,
Filip De Turck, Femke Ongenae

This additional file discusses the PoC implementation of the use case demonstrator. It provides details about the configuration of the different building blocks in the demonstrator architecture in Figure 5 of the main paper. In the implementation, the domain knowledge, context information of Rosa and sensor observations are semantically annotated using an extended version of the ACCIO continuous care ontology [1, 2].

1 RMLStreamer

The RMLStreamer maps each observation in the JSON input stream to an observation in the RDF output stream. To this end, concepts and relations defined in the extended version of the ACCIO ontology are used. An example JSON observation is shown in Listing 1. The resulting RDF observation after mapping it with RML mapping rules is shown in Listing 2.

To define how the mapping should be performed by the RMLStreamer, an RML mapping file needs to be configured. The RML mapping file used in the PoC implementation of the use case demonstrator is presented in Listing 3. The last part of this mapping file (lines 73–77) defines the input stream for the RMLStreamer: when started, a job is started on an Apache Flink cluster which opens a connection to a TCP socket stream on a certain host and port to pull the incoming messages from the stream. The semantically annotated sensor observations in RDF are then pushed by this job on a TCP socket output stream on a user-defined port.

2 DIVIDE and C-SPARQL

The contextually relevant C-SPARQL queries are derived and configured by DIVIDE when the use case context associated to patient Rosa is updated. To derive specific queries in DIVIDE, generic versions of these queries need to be loaded into the system. Based on the context and domain knowledge, the DIVIDE query derivation can then

Listing 1: Example JSON input data file that can be mapped to the RDF data in Listing 2 using the RML mapping file in Listing 3

```
{
  "observations": [
    {
      "id": "123e4567-e89b-12d3-a456-556642440000",
      "observedProperty": "PersonStep",
      "madeBySensor": "c1-19-24-70-fb-6d-S2",
      "time": "2023-04-17T14:48:22.850Z",
      "value": 1
    }
  ]
}
```

Listing 2: Example sensor observation in RDF/Turtle syntax, represented in the ACCIO continuous care ontology, which is the result of mapping the example JSON input data file in Listing 1 using the RML mapping file in Listing 3

```
@prefix entity: <http://occs.intec.ugent.be/ontology/entity#> .
@prefix obs: <http://occs.intec.ugent.be/ontology/observations#> .
@prefix rdf: <http://www.w3.org/1999/02/22-rdf-syntax-ns#> .
@prefix xsd: <http://www.w3.org/2001/XMLSchema#> .
@prefix sosa: <http://www.w3.org/ns/sosa/> .
@prefix General: <http://IBCNServices.github.io/Accio-Ontology/General.owl#> .
@prefix SSNiot: <http://IBCNServices.github.io/Accio-Ontology/SSNiot.owl#> .
@prefix DUL: <http://IBCNServices.github.io/Accio-Ontology/ontologies/DUL.owl#> .

obs:Observation_123e4567-e89b-12d3-a456-556642440000
    rdf:type sosa:Observation ;
    General:hasId [ General:hasID "123e4567-e89b-12d3-a456-556642440000"^^xsd:
         string ] ;
    sosa:observedProperty [ rdf:type SSNiot:PersonStep ] ;
    sosa:madeBySensor entity:c1-19-24-70-fb-6d-S2 ;
    sosa:resultTime "2023-04-17T14:48:22.850Z"^^xsd:dateTime ;
    sosa:hasResult [
        DUL:hasDataValue "1"^^xsd:float ;
    ] .
```

perform reasoning to derive for which sensors these queries need to be instantiated, and how this instantiation should happen.

The ACCIO ontology makes use of an observation pattern involving the classes `Observation`, `Symptom`, `Fault`, `Action` and `Alarm`. Figure 1 details how these classes are linked. Moreover, it gives an example of a series of subclasses that model a symptom, fault, action and alarm related to an observation of the `BodyTemperature` property that has a value exceeding a certain, medically defined threshold. This is especially relevant to understand how the query is derived that filters Rosa's fever events when her medical profile is updated with the colon cancer diagnosis.

Figure 2 details how the diagnoses occurring in the use case scenario, dementia and colon cancer, are semantically modeled in the extension of the ACCIO continuous care ontology, according to the medical knowledge owned by the hospital about these diseases. These definitions are used by DIVIDE to convert the generic DIVIDE queries to the specific C-SPARQL queries of the use case scenario. Three generic DIVIDE queries

Listing 3: RML mapping file used by the RMLStreamer in the PoC implementation of the use case demonstrator, presented in RDF/Turtle syntax (part 1/2). `[...]` is a placeholder for omitted parts that are not of interest.

```
@prefix rr: <http://www.w3.org/ns/r2rml#>.
@prefix rml: <http://semweb.mmlab.be/ns/rml#>.
@prefix rmls: <http://semweb.mmlab.be/ns/rmls#> .
@prefix rdf: <http://www.w3.org/1999/02/22-rdf-syntax-ns#>.
@prefix rdfs: <http://www.w3.org/2000/01/rdf-schema#>.
@prefix ql: <http://semweb.mmlab.be/ns/ql#>.
@prefix map: <http://mapping.example.com/>.

# defines which triple subjects should be linked to the different predicate-object maps
map:map_observations_0 rml:logicalSource map:source; a rr:TriplesMap;
    rdfs:label "observations"; rr:subjectMap map:s_0;
    rr:predicateObjectMap map:pom_0, map:pom_1, map:pom_2, map:pom_3,
                          map:pom_4, map:pom_5.
map:map_ids_0 rml:logicalSource map:source; a rr:TriplesMap;
    rdfs:label "ids"; rr:subjectMap map:s_1; rr:predicateObjectMap map:pom_6.
map:map_props_0 rml:logicalSource map:source; a rr:TriplesMap;
    rdfs:label "props"; rr:subjectMap map:s_2; rr:predicateObjectMap map:pom_7 .
map:map_results_0 rml:logicalSource map:source; a rr:TriplesMap;
    rdfs:label "results"; rr:subjectMap map:s_3; rr:predicateObjectMap map:pom_8.

# define the object maps (which entities and datatypes should be present in
#                         the objects of the 9 resulting RDF triples)
map:om_0 a rr:ObjectMap; rr:termType rr:IRI;
    rr:constant "http://www.w3.org/ns/sosa/Observation".
map:om_1 a rr:ObjectMap; rr:termType rr:Literal;
    rr:template "http://occs.intec.ugent.be/ontology/observations#Observation_{id}_id".
map:om_2 a rr:ObjectMap; rr:termType rr:Literal;
    rr:template "http://occs.intec.ugent.be/ontology/observations#Observation_{id}_prop".
map:om_3 a rr:ObjectMap; rr:termType rr:Literal;
    rr:template "http://occs.intec.ugent.be/ontology/entity#{madeBySensor}".
map:om_4 a rr:ObjectMap; rml:reference "time"; rr:termType rr:Literal;
    rr:datatype <http://www.w3.org/2001/XMLSchema#datetime>.
map:om_5 a rr:ObjectMap; rr:termType rr:Literal; rr:template
    "http://occs.intec.ugent.be/ontology/observations#Observation_{id}_result".
map:om_6 a rr:ObjectMap; rml:reference "id"; rr:termType rr:Literal;
    rr:datatype <http://www.w3.org/2001/XMLSchema#string>.
map:om_7 a rr:ObjectMap; rr:termType rr:IRI; rr:template
    "http://IBCNServices.github.io/Accio-Ontology/SSNiot.owl#{observedProperty}".
map:om_8 a rr:ObjectMap; rml:reference "value"; rr:termType rr:Literal;
    rr:datatype <http://www.w3.org/2001/XMLSchema#double>.

# define the predicate maps
# (which predicates should be used in the 9 resulting RDF triples)
map:pm_0 a rr:PredicateMap; rr:constant rdf:type.
map:pm_1 a rr:PredicateMap;
    rr:constant <http://IBCNServices.github.io/Accio-Ontology/General.owl#hasId>.
map:pm_2 a rr:PredicateMap; rr:constant <http://www.w3.org/ns/sosa/observedProperty>.
map:pm_3 a rr:PredicateMap; rr:constant <http://www.w3.org/ns/sosa/madeBySensor>.
map:pm_4 a rr:PredicateMap; rr:constant <http://www.w3.org/ns/sosa/resultTime>.
map:pm_5 a rr:PredicateMap; rr:constant <http://www.w3.org/ns/sosa/hasResult>.
map:pm_6 a rr:PredicateMap;
    rr:constant <http://IBCNServices.github.io/Accio-Ontology/General.owl#hasID>.
map:pm_7 a rr:PredicateMap; rr:constant rdf:type.
map:pm_8 a rr:PredicateMap;
    rr:constant <http://IBCNServices.github.io/Accio-Ontology/ontologies/DUL.owl#hasDataValue>.

# link the predicates to the objects in a predicate-object map
map:pom_0 a rr:PredicateObjectMap; rr:predicateMap map:pm_0; rr:objectMap map:om_0.
[...]
map:pom_8 a rr:PredicateObjectMap; rr:predicateMap map:pm_8; rr:objectMap map:om_8.
```

Listing 3: RML mapping file used by the RMLStreamer in the PoC implementation of the use case demonstrator, presented in RDF/Turtle syntax (part 2/2)

```
# define subject maps (containing templates representing which entities should be
#                      used in the subjects of the 9 resulting triples)
map:s_0 a rr:SubjectMap; rr:template
    "http://occs.intec.ugent.be/ontology/observations#Observation_{id}".
map:s_1 a rr:SubjectMap; rr:template
    "http://occs.intec.ugent.be/ontology/observations#Observation_{id}_id".
map:s_2 a rr:SubjectMap; rr:template
    "http://occs.intec.ugent.be/ontology/observations#Observation_{id}_prop".
map:s_3 a rr:SubjectMap; rr:template
    "http://occs.intec.ugent.be/ontology/observations#Observation_{id}_result".

# define source (input data stream) for RMLStreamer to read from
map:source a rml:LogicalSource;
    rml:source [ rdf:type rmls:TCPSocketStream ;
                 rmls:hostName "192.168.1.49";
                 rmls:type "PULL" ; rmls:port "5005" ];
    rml:referenceFormulation ql:JSONPath.
```

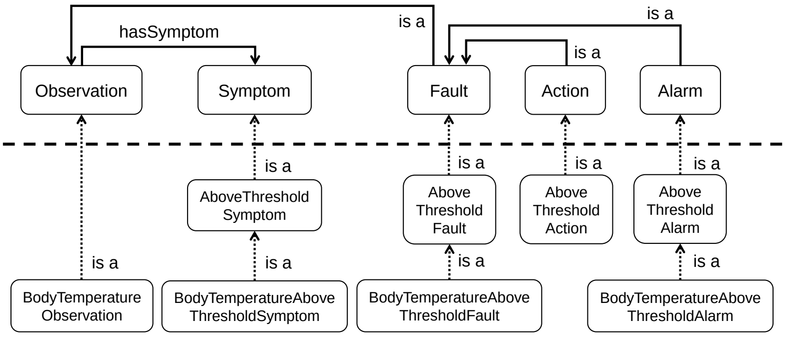

Figure 1: Observation pattern in the ACCIO continuous care ontology. This pattern is used in the use case demonstrator in the DIVIDE query that yields the C-SPARQL query filtering high body temperature (fever) events.

are configured in the PoC implementation of the demonstrator. This follows from the diagnosis overview in Figure 2: there is one DIVIDE query corresponding to the medical symptom associated with colon cancer, and one DIVIDE query corresponding to each requirement associated to the dementia diagnosis.

Listing 4 presents the sensor query rule with generic query pattern of the first DIVIDE query that filters `AboveThresholdAlarm` instances. This is a subclass of the `Alarm` class of the observation pattern of the ACCIO ontology, as shown in Figure 1. For an `Observation` to also be an `AboveThresholdAlarm`, some conditions must be fulfilled. One of these conditions is that the `Observation` is linked to an `AboveThresholdSymptom`. The sensor query rule in Listing 4 links an `Observation` to an `AboveThresholdSymptom` if a threshold `?threshold` is crossed. Through reasoning, DIVIDE will only instantiate this rule, and hence the generic query pattern, for the

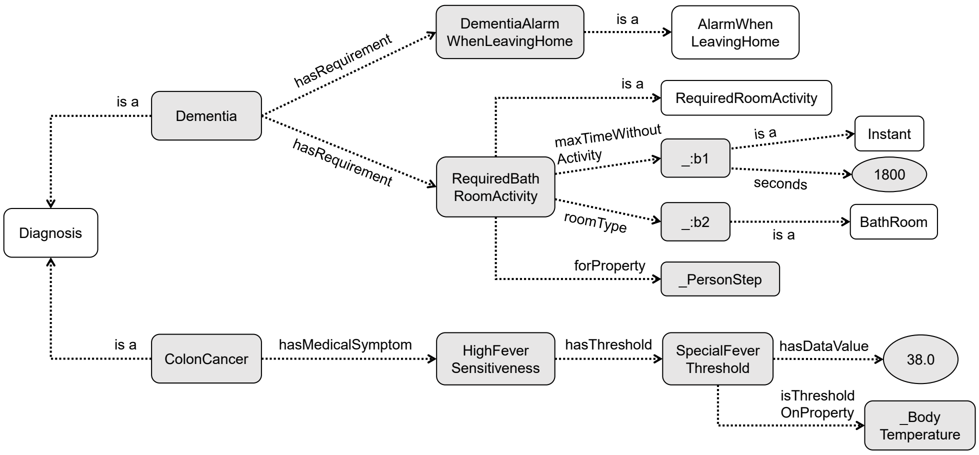

Figure 2: Overview of how diagnoses are modeled in the medical domain knowledge. This is shown for the diagnoses occurring in the demonstrator's use case scenario. The modeling is performed using the extended version of the ACCIO ontology. For readability purposes, ontology prefixes are omitted.

cases where the `Observation` with an `AboveThresholdSymptom` is also an instance of `AboveThresholdAlarm`. This happens through the semantic reasoning. In Rosa's case, this query will instantiate for `?prop` being `SSNiot:BodyTemperature` when her medical profile contains the triple:

```
:Rosa CareRoomMonitoring:hasDiagnosis CareRoomMonitoring:ColonCancer .
```

The other two DIVIDE queries corresponding to the dementia diagnosis are similar to the presented DIVIDE query. However, they do not use the generic ontology observation pattern, but directly model the requirements associated to the dementia diagnosis shown in Figure 2.

3 Streaming MASSIF

In the PoC implementation of the use case demonstrator, the different layers of Streaming MASSIF are employed. The configuration details of each layer are discussed below.

- The selection layer uses the C-SPARQL engine as described before, which continuously evaluates the queries derived by DIVIDE.
- The abstraction layer of Streaming MASSIF abstracts the body temperature sensor events filtered by C-SPARQL to high-level events. This is done through expressive semantic reasoning, using the rules explained in the description of step 4 of the demonstrator's use case scenario. As an example, consider the following high-level definition to describe a *medium fever event*:

```
MediumTemperatureEvent =
    AboveTemperatureThresholdAlarm and
    hasResult some (
        (hasDataValue some xsd:double[>= "38.5"^^xsd:double]) and
```

Listing 4: Sensor query rule with the generic query pattern of the DIVIDE query in the use case demonstrator that filters instances of the `AboveThresholdAlarm` class (part 1/2)

```
@prefix : <http://idlab.ugent.be/sensdesc/query#> .
@prefix sd: <http://idlab.ugent.be/sensdesc#> .
@prefix sh: <http://www.w3.org/ns/shacl#> .
@prefix owl: <http://www.w3.org/2002/07/owl#> .
@prefix rdf: <http://www.w3.org/1999/02/22-rdf-syntax-ns#> .
@prefix xsd: <http://www.w3.org/2001/XMLSchema#> .
@prefix ssn: <http://www.w3.org/ns/ssn/> .
@prefix rdfs: <http://www.w3.org/2000/01/rdf-schema#> .
@prefix sosa: <http://www.w3.org/ns/sosa/> .
@prefix DUL: <http://IBCNServices.github.io/Accio-Ontology/ontologies/DUL.owl#> .
@prefix SSNiot: <http://IBCNServices.github.io/Accio-Ontology/SSNiot.owl#> .
@prefix RoleCompetenceAccio:
    <http://IBCNServices.github.io/Accio-Ontology/RoleCompetenceAccio.owl#> .
@prefix CareRoomMonitoring:
    <http://IBCNServices.github.io/Accio-Ontology/CareRoomMonitoring.owl#> .

{
    ?p DUL:hasRole [ rdf:type RoleCompetenceAccio:PatientRole ] ;
       DUL:hasLocation ?l ;
       CareRoomMonitoring:hasDiagnosis [
           CareRoomMonitoring:hasMedicalSymptom [
               rdf:type CareRoomMonitoring:HighSensitivity ;
               SSNiot:hasThreshold [
                   DUL:hasDataValue ?threshold ;
                   SSNiot:isThresholdOnProperty [ rdf:type ?prop ]
               ]
           ]
       ] .

    ?sensor rdf:type sosa:Sensor ;
            sosa:observes [ rdf:type ?prop ] ;
            SSNiot:isSubsystemOf [ DUL:hasLocation ?l ] .

    ?prop rdfs:subClassOf sosa:ObservableProperty .
} => {
    _:q rdf:type sd:Query ;
        sd:pattern :pattern-above-threshold-alarm ;
        sd:inputVariables (("?prop" ?prop) ("?threshold" ?threshold)
                           ("?sensor" ?sensor) ("?patient" ?p)) ;
        sd:outputVariables (("?v" _:v) ("?o" _:oo)) .

    _:oo rdf:type sosa:Observation ;
         sosa:madeBySensor ?sensor ;
         sosa:hasResult [
             rdf:type SSNiot:QuantityObservationValue ;
             DUL:hasDataValue _:v ] ;
         SSNiot:hasSymptom [
             rdf:type CareRoomMonitoring:AboveThresholdSymptom ;
             ssn:forProperty [ rdf:type ?prop ] ] .
} .

:prefixes-above-threshold-alarm rdf:type owl:Ontology ;
    sh:declare [ sh:prefix "xsd" ;
                 sh:namespace "http://www.w3.org/2001/XMLSchema#"^^xsd:anyURI ] ;
    sh:declare [ sh:prefix "ssn" ; sh:namespace "http://www.w3.org/ns/ssn/"^^xsd:
        anyURI ] ;
    sh:declare [ sh:prefix "sosa" ;
                 sh:namespace "http://www.w3.org/ns/sosa/"^^xsd:anyURI ] ;
    sh:declare [ sh:prefix "General" ; sh:namespace "http://IBCNServices.github.io/
        Accio-Ontology/General.owl#"^^xsd:anyURI ] ;
    sh:declare [ sh:prefix "CareRoomMonitoring" ; sh:namespace "http://IBCNServices
        .github.io/Accio-Ontology/CareRoomMonitoring.owl#"^^xsd:anyURI ] ;
    sh:declare [ sh:prefix "DUL" ; sh:namespace "http://IBCNServices.github.io/
        Accio-Ontology/ontologies/DUL.owl#"^^xsd:anyURI ] .
```

Listing 4: Sensor query rule with the generic query pattern of the DIVIDE query in the use case demonstrator that filters instances of the `AboveThresholdAlarm` class (part 2/2)

```
:pattern-above-threshold-alarm
    rdf:type sd:QueryPattern ;
    sh:prefixes :prefixes-above-threshold-alarm ;
    sh:construct """
        CONSTRUCT {
            ?o a CareRoomMonitoring:AboveThresholdAlarm ;
               ssn:forProperty ?prop ;
               DUL:associatedWith ?patient ;
               sosa:hasResult [ DUL:hasDataValue ?v ] .
        }
        FROM NAMED WINDOW :win ON <http://idlab.ugent.be/grove> [RANGE PT5S SLIDE
             PT3S]
        WHERE {
            WINDOW :win {
                ?o a sosa:Observation ;
                   sosa:madeBySensor ?sensor ;
                   sosa:hasResult [ DUL:hasDataValue ?v ] ;
                   sosa:resultTime ?t ;
                   General:hasId [ General:hasID ?id ] .
                FILTER (xsd:double(?v) > xsd:double(?threshold))
            }
        }
        ORDER BY DESC(?t)
        LIMIT 1
        """ .
```

```
                  (hasDataValue some xsd:double[< "39"^^xsd:double]))
```

The definitions to describe a *low fever event* and *high fever event* are completely similar.

- Streaming MASSIF's temporal reasoning layer detects temporal dependencies between high-level events. The high-level definition of a *rising fever event* is semantically described as follows:

```
RisingTemperatureEvent =
    every (a=LowTemperatureEvent -> b=MediumTemperatureEvent
           -> c=HighTemperatureEvent) where timer:within(3600 sec)
```

On top of Streaming MASSIF's temporal reasoning layer, two queries are defined for the instructed notification service. The query representing the rule to send a caregiver with a scheduled visit to the patient in case of a *low fever event*, is shown in Listing 5. The other query processing any *rising fever event* is very similar.

4 AMADEUS

To compose a workflow representing a treatment plan to Rosa's colon cancer, AMADEUS starts from the current state defined in the knowledge base. In the demonstrator use case, this RDF state description contains Rosa's personal information, and medical information: diagnosis, tumor size, risk of metastasis, etc. An example of this state description is shown in Listing 6. To represent the medical diagnoses, the implementation makes use of the Systematized Nomenclature of Medicine Clinical Terms (SNOMED CT) [3].

Listing 5: Query of Streaming MASSIF's instructed notification service that generates a notification to a caregiver with a scheduled patient visit in case of a *low fever event*

```
PREFIX rdf: <http://www.w3.org/1999/02/22-rdf-syntax-ns#>
PREFIX owl: <http://www.w3.org/2002/07/owl#>
PREFIX rdfs: <http://www.w3.org/2000/01/rdf-schema#>
PREFIX xsd: <http://www.w3.org/2001/XMLSchema#>
PREFIX DUL: <http://IBCNServices.github.io/Accio Ontology/ontologies/DUL.owl#>
PREFIX role: <http://ibcnservices.github.io/Accio Ontology/RoleCompetenceAccio.owl
    #>
PREFIX : <http://idlab.dissect.healthdemo/selectionservice.owl#>

CONSTRUCT { ?visitor rdf:type :VisitorLowPriority}
WHERE {
    ?fever rdf:type :LowTemperatureEvent.
    ?fever DUL:associatedWith ?patient.
    ?patient :hasSchedule ?schedule.
    ?schedule :hasDaySchedule :todaysSchedule.
    :todaysSchedule :hasItem ?item.
    ?item :hasVisitor ?visitor.
    ?visitor DUL:hasRole ?role.
    ?role rdf:type role:Child.
    FILTER NOT EXISTS { ?rising rdf:type :RisingTemperatureEvent }
}
```

When running the EYE reasoner, a goal should be defined to represent what the target state is that EYE should be looking for when composing workflows. An example of the goal description for the demonstrator use case is shown in Listing 7. Moreover, the inputs of the EYE reasoner contain different policies as a Weighted Transition Logic in N3 with medical domain knowledge about treating colon cancer. These policies are essentially step descriptions, describing the changes they will make to the state description. Listing 8 contains the example of a colon cancer policy representing the possible surgery step in colon cancer treatment. Listing 9 contains some examples of additionally relevant medical domain knowledge: preconditions that allow a patient to take surgery, rules to calculate the relapse risk after surgery for different situations, and definitions of contraindications that can result in conflicting workflows.

References

[1] Ongenae, F., Duysburgh, P., Sulmon, N., Verstraete, M., Bleumers, L., De Zutter, S., Verstichel, S., Ackaert, A., Jacobs, A., De Turck, F.: An ontology co-design method for the co-creation of a continuous care ontology. Applied Ontology **9**(1), 27–64 (2014) https://doi.org/10.3233/AO-140131

[2] De Brouwer, M., Ongenae, F., Bonte, P., De Turck, F.: Towards a Cascading Reasoning Framework to Support Responsive Ambient-Intelligent Healthcare Interventions. Sensors **18**(10), 3514 (2018) https://doi.org/10.3390/s18103514

[3] Donnelly, K.: SNOMED-CT: The advanced terminology and coding system for eHealth. Studies in Health Technology and Informatics **121** (2006)

Listing 6: Initial state description used by AMADEUS in the use case demonstrator to compose medical treatment plans to treat colon cancer, in N3 syntax

```
PREFIX sct: <http://snomed.info/id/>
PREFIX data: <https://gitlab.ilabt.imec.be/KNoWS/dissect/data#>
PREFIX care: <https://gitlab.ilabt.imec.be/KNoWS/dissect/care#>

# Rosa's patient data
data:patient_1 a care:Patient.
data:patient_1 care:age 74 .
data:patient_1 care:name "Rosa" .
data:patient_1 care:gender "female" .
data:patient_1 care:weight 63 .

# colon cancer diagnosis
data:patient_1 care:diagnosis sct:363406005, sct:363351006 .
data:patient_1 care:tumor_size 40 .
data:patient_1 care:metastasis_risk 0.4 .
data:patient_1 care:5yr_survival_rate 0.2 .
data:patient_1 care:non_toxicity 1 .
data:patient_1 care:position sct:34402009 .
data:patient_1 care:status "active" .
data:patient_1 care:tnm_t 3 .
data:patient_1 care:blocking_colon false .
data:patient_1 care:5yr_local_relapse_risk 0 .
```

Listing 7: Goal description used by AMADEUS in the use case demonstrator to compose medical treatment plans to treat colon cancer, in N3 syntax

```
PREFIX math: <http://www.w3.org/2000/10/swap/math#>
PREFIX xsd: <http://www.w3.org/2001/XMLSchema#>
PREFIX gps: <http://josd.github.io/eye/reasoning/gps/gps-schema#>
PREFIX sct: <http://snomed.info/id/>
PREFIX care: <https://gitlab.ilabt.imec.be/KNoWS/dissect/care#>

{
    ?SCOPE gps:findpath (
    {
        ?patient a care:Patient.
        ?patient care:diagnosis sct:363406005.
        ?patient care:tumor_size 0 .
        ?patient care:metastasis_risk ?risk .
        ?patient care:5yr_survival_rate ?rate .
        ?patient care:non_toxicity ?non_toxicity .
        ?patient care:5yr_local_relapse_risk ?relapse_risk .

        # additional requirements for the treatment plan could be defined as shown
            below
        # ?risk math:lessThan 0.1 .
        # ?rate math:greaterThan 0.7 .
        # ?non_toxicity math:greaterThan 0.5 .
        # ?relapse_risk math:lessThan 0.15 .
    }
    ?PATH ?DURATION ?COST ?BELIEF ?COMFORT
    ("P150D"^^xsd:dayTimeDuration 200000.0 0.1 0.1)).
} => {
    ?patient gps:path (?PATH ?DURATION ?COST ?BELIEF ?COMFORT
                       (?risk ?rate ?non_toxicity ?relapse_risk)).
} .
```

Listing 8: Example of a colon cancer policy (surgery step description) used by AMADEUS in the use case demonstrator to compose medical treatment plans to treat colon cancer, in N3 syntax

```
PREFIX math: <http://www.w3.org/2000/10/swap/math#>
PREFIX xsd: <http://www.w3.org/2001/XMLSchema#>
PREFIX e: <http://eulersharp.sourceforge.net/2003/03swap/log-rules#>
PREFIX gps: <http://josd.github.io/eye/reasoning/gps/gps-schema#>
PREFIX action: <https://gitlab.ilabt.imec.be/KNoWS/dissect/action#>
PREFIX sct: <http://snomed.info/id/>
PREFIX surgery: <https://gitlab.ilabt.imec.be/KNoWS/dissect/surgery#>
PREFIX care: <https://gitlab.ilabt.imec.be/KNoWS/dissect/care#>

# surgery step description
{
    care:Colon_cancer gps:description (
        {
            ?patient care:tumor_size ?size.
            ?patient care:metastasis_risk ?risk .
            ?patient care:5yr_survival_rate ?rate .
            ?patient care:non_toxicity ?non_toxicity .
            ?patient care:5yr_local_relapse_risk ?relapse_risk .
        }
        { ?patient gps:surgery surgery:surgery_colon_cancer. }
        {
            # surgery should completely remove the tumor
            ?patient care:tumor_size 0 .
            ?patient care:metastasis_risk ?new_risk .
            ?patient care:5yr_survival_rate ?new_rate .
            ?patient care:non_toxicity ?new_non_toxicity .
            ?patient care:taken action:surgery_colon_cancer.
            ?patient care:5yr_local_relapse_risk ?new_relapse_risk .
        }
        action:surgery_colon_cancer
        # defines duration, cost, belief & comfort quality parameters of surgery
        "P5D"^^xsd:dayTimeDuration 20950 0.9 0.5
    )
} <= {
    ?patient a care:Patient.
    ?patient care:diagnosis sct:363406005.
    ?patient care:surgery_colon_cancer_precondition true .
    ?scope e:fail { ?patient care:taken action:surgery_colon_cancer. }.

    ?patient care:post_surgery_5yr_local_relapse_risk ?new_relapse_risk .

    ?patient care:metastasis_risk ?risk .
    (?risk 0.1) math:product ?new_risk.

    # surgery decreases the 5 year death rate (= 1 - 5 year survival rate) with 80%
    ?patient care:5yr_survival_rate ?rate .
    (1 ((1 ?rate)!math:difference 0.2)!math:product) math:difference ?new_rate.

    ?patient care:non_toxicity ?non_toxicity .
    (?non_toxicity 0.95) math:product ?new_non_toxicity.
}.
```

Listing 9: Examples of additionally relevant medical domain knowledge, used by AMADEUS in the use case demonstrator to compose medical treatment plans to treat colon cancer and automatically detect conflicts between treatment plans, in N3 syntax

```
PREFIX math: <http://www.w3.org/2000/10/swap/math#>
PREFIX action: <https://gitlab.ilabt.imec.be/KNoWS/dissect/action#>
PREFIX sct: <http://snomed.info/id/>
PREFIX therapy: <https://gitlab.ilabt.imec.be/KNoWS/dissect/therapy#>
PREFIX care: <https://gitlab.ilabt.imec.be/KNoWS/dissect/care#>
PREFIX medication: <https://gitlab.ilabt.imec.be/KNoWS/dissect/medication#>

# preconditions that allow a patient to take surgery
{
    ?patient care:surgery_colon_cancer_precondition true .
} <= {
    ?patient a care:Patient.
    ?patient care:diagnosis sct:363406005, sct:363351006.
    ?patient care:tnm_t ?t_value .
    ?t_value math:lessThan 3 .
}.
{
        ?patient care:surgery_colon_cancer_precondition true .
} <= {
        ?patient a care:Patient.
        ?patient care:diagnosis sct:363406005, sct:363351006.
        ?patient care:tnm_t ?t_value .
        ?t_value math:greaterThan 2 .
        ?patient care:taken action:Neoadjuvant_chemoradiotherapy.
}.

# rules to calculate the relapse risk after surgery for different situations
{
    ?patient care:post_surgery_5yr_local_relapse_risk 0.04 .
} <= {
    ?patient care:tnm_t 2 .
}.
{
    ?patient care:post_surgery_5yr_local_relapse_risk 0.06 .
} <= {
    ?patient care:taken action:Neoadjuvant_chemoradiotherapy.
    ?patient care:tnm_t 3 .
}.

# definitions of contraindications that can result in conflicting workflows
# -> patient has influenza
{
    ?patient therapy:hasContraindicationForChemotherapy true
} <= {
    ?patient a care:Patient.
    ?patient care:diagnosis sct:6142004. # influenza diagnosis
}.
# -> or patient takes medications that conflict with chemoradiotherapy medications
{
    ?patient therapy:hasContraindicationForChemotherapy true
} <= {
    ?patient a care:Patient.
    ?patient therapy:medication ?med.
    ?med medication:contraindication therapy:chemoradiotherapy.
}.
```
